# Design, Synthesis and Biological Evaluation of Novel Benzothiazole Derivatives as Selective PI3Kβ Inhibitors

**DOI:** 10.3390/molecules21070876

**Published:** 2016-07-02

**Authors:** Shuang Cao, Ruiyuan Cao, Xialing Liu, Xiang Luo, Wu Zhong

**Affiliations:** 1 Laboratory of Computer-Aided Drug Design & Discovery, Beijing Institute of Pharmacology and Toxicology, Beijing 100850, China; caoshuangknight@163.com (S.C.); 21cc@163.com (R.C.); 2 Hubei Key Laboratory of Novel Chemical Reactor and Green Chemical Technology, Wuhan Institute of Technology, Wuhan 430073, Hubei, China; liuxialing@hotmail.com (X.L.); rosen_luo@163.com (X.L.)

**Keywords:** PI3Kβ, mTOR, kinase inhibitor, docking

## Abstract

A novel series of PI3Kβ (Phosphatidylinositol-3-kinases beta subunit) inhibitors with the structure of benzothiazole scaffold have been designed and synthesized. All the compounds have been evaluated for inhibitory activities against PI3Kα, β, γ, δ and mTOR (Mammalian target of rapamycin). Two superior compounds have been further evaluated for the IC_50_ values against PI3Ks/mTOR. The most promising compound **11** displays excellent anti-proliferative activity and selectivity in multiple cancer cell lines, especially in the prostate cancer cell line. Docking studies indicate the morpholine group in 2-position of benzothiazole is necessary for the potent antitumor activity, which confirms our design is reasonable.

## 1. Introduction

PI3Ks (phosphatidylinositol-3-kinases) are a group of cellular signaling molecules, which play a critical role in cell growth, proliferation, motility and survival [1]. Dysregulation of the PI3Ks signaling pathway is significantly associated with human cancers. According to the structural characteristics and substrate preferences, the PI3K family can be divided into three classes (I, II and III) [2]. Class I of PI3Ks has been further classified into Class IA, comprising PI3Kα, β and δ catalytic subunits, and Class IB (PI3Kγ catalytic subunit) [3]. PI3Kα and β are expressed in most normal tissues, whereas PI3Kδ and γ are thought to be restricted to the immune system. Though all the four catalytic subunits have a function of generating phosphatidylinositol 3,4,5-triphosphate (PIP3), a second messenger involved in Akt activation, most solid tumor cells just express PI3Kα and PI3Kβ [4]. For the reasons above, PI3Kα and β are considered as the more appropriate treatment target of cancer.

PI3Kα has been confirmed as one of the most common mutated genes identified in human cancers, which has been drawing considerable attention for years. PI3Kβ was previously thought to be involved in regulating the formation of arterial thrombi [5] and had a slight effect in the development of tumors; however, new findings have confirmed that PI3Kβ shows remarkable overexpression in some special tumors, such as the tumors losing of PTEN (Phosphatase and tensin homolog deleted on chromosome ten). In animal models, genetic approaches have demonstrated that PI3Kβ but not PI3Kα activation is required for the development of PTEN-deletion tumors [2,6,7,8,9,10]. For example, it has been reported that overexpression of PI3Kβ induces early lesions of prostatic tumor (a typical PTEN-deficient tumor) formation in mice [1,2]. In addition, PI3Kβ also shows considerable overexpression in some chemo-resistant cancer cell lines [11]. All these new discoveries hint at the significant and particular role of PI3Kβ in the development of some special cancers [12] and also inspire us to consider that inhibiting the activity of PI3Kβ would be more effective than PI3Kα in the treatment of some PTEN-deficient tumors and chemo-resistant tumors, such as in prostate cancer. Therefore, PI3Kβ inhibitors have the potential to become a new generation of anti-prostate cancer drugs.

Most of the early PI3K inhibitors belong to pan-PI3K inhibitors, which inhibit all four PI3K Class I subunits. Nearly all of these pan-PI3K inhibitors have an excellent effect on blocking all class I PI3Ks, while the draw backs are also obvious. Compounds targeting all class I PI3Ks may not be tolerated. Furthermore, pan-PI3K inhibitors commonly have off-target effects on members of the PI3K-related kinase (PIKK) family (mTOR, DNA-PK, ATM, ATR) and other cell components [1]. Inhibition of specific PI3K subunit can provide a strategy of maximizing the potential therapeutic window. At the same time, this strategy can be applied as accurate therapy to various types of cancers alone or in combination with other targeted therapies. Compared with the pan-PI3K inhibitors, specific PI3K inhibitors are reported less, especially the PI3Kβ inhibitors. Because there are only subtle structural differences among the four PI3K subunits, and the knowledge on the function of PI3Kβ was insufficient in the past. Encouragingly, some subtle differences between the four subunits have been found not only in protein structures but also in functions in recent years. Alongside such findings, some isoform-selective PI3K inhibitors begin to emerge. 

Given the importance and scarcity of PI3Kβ inhibitors, a series of PI3Kβ-selective inhibitors are developed and their potential applications as anticancer agents in tumor models are reported herein.

**Figure 1:**
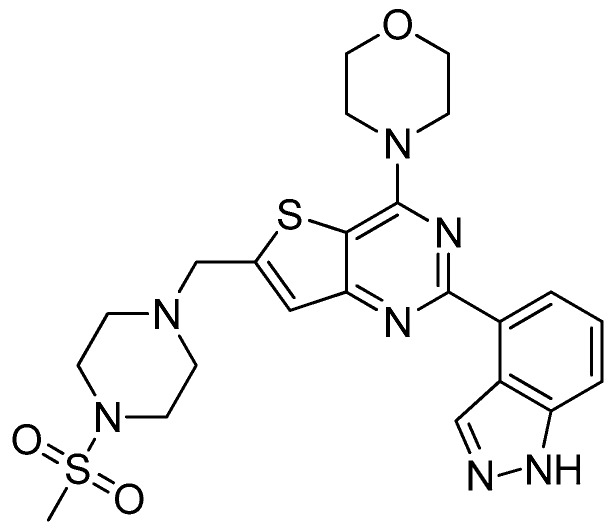
Structure of GDC-0941.

**Figure 2:**
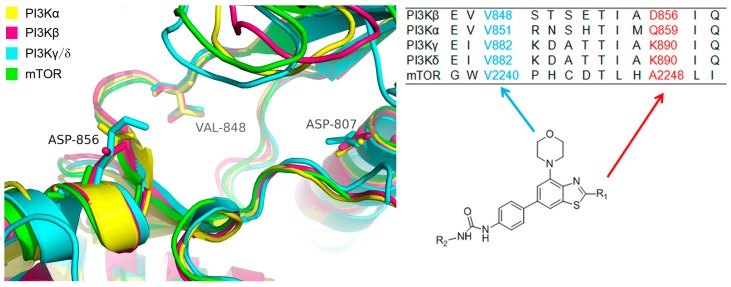
(**Left**): structure superimposed of PI3Kα, β, γ, δ and mTOR. Red: PI3Kβ (4BFR), yellow: PI3Kα (3ZIM), blue: PI3Kγ (5EDS) or PI3Kδ (4GB9), green: mTOR (4JT6); (**Right**): part of the sequence alignment of PI3Ks and mTOR, and the corresponding docking position in newly designed compounds.

PI3Kα, β, γ and δ share the similar sequence and 3D protein structure in the ATP-binding site (such as VAL848 and ASP807 in PI3Kβ), which explains that the pan-PI3K inhibitor can inhibit all PI3K subunits. However, some amino acid residues are still different. Sequence alignment of ATP-binding sites of PI3Kα, β, γ and δ shows that ASP856 in PI3Kβ is unique. Considering the aspartic acid is an ideal hydrogen bond donor and the appropriate spatial distance between ASP856 and VAL848, a proper group is planned to introduce to the newly designed compounds which makes them specifically interact with ASP856. After the specific point is determined, the new compounds can be formulated gradually. GDC-0941 [13], a pan-PI3K inhibitor with representative structure and extremely high potency, was selected as the template to design the new PI3Kβ isoform-selective inhibitor (Figure 1). VAL848, ASP807 and LYS799 were identified as the essential residues in interaction between inhibitor and the PI3Ks Class I, through analyzing the GDC-0941 binding pocket in cocrystal of PI3Ks (Figure 2). Maintaining interaction with these key residues is also the point in our compound design.

Based on these analyses, the strategies in this paper were reserving the key groups in GDC-0941, such as the morpholine group (interacting with VAL848) and carbamide group (interacting with ASP807 and LYS799), modifying the thieno[3,2-*d*]pyrimidine (scaffold structure of GDC-0941) and introducing a specific group bonding with ASP856 to improve the selectivity of inhibitors (Figure 2). Then, the benzothiazole was chosen as scaffold structure, and different groups including hydrogen bond donor and acceptor were introduced to the 2-positon of benzothiazole to form a hydrogen bond with ASP856 in PI3Kβ. Finally, a series of substituents were linked into the terminal of carbamide group to make an additional interaction with PI3Kβ. The synthesis, biological activity and selectivity assays of the compounds were presented herein.

## 2. Results and Discussion

### 2.1. Chemistry

**Scheme 1:**
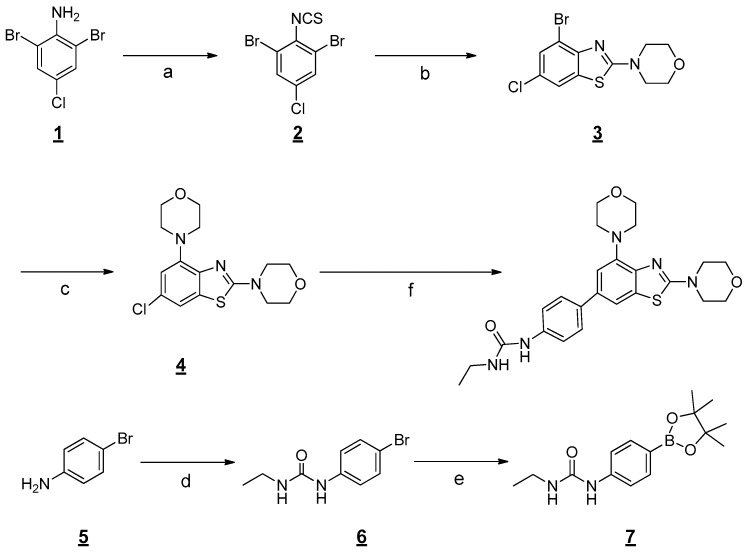
Synthesis route of compound **1**. *Reactions and conditions*: (**a**) Thiophosgene, 4-dimethylaminopyridine (DMAP), CH_2_Cl_2_, 40 °C, 6–8 h, 95%; (**b**) 1. Morpholine, dioxane, 25 °C, 0.5 h; 2. DMAP, CsCO_3_, dioxane, 105 °C, 3 h, 92%; (**c**) Morpholine, Pd_2_(dba)_3_, CsCO_3_, (*R*)-(+)-2,2′-Bis(diphenylphosphino)-1,1′-binaphthalene (R-BINAP), DMF, 90 °C, 8 h, 82%; (**d**) Isocyanatoethane, CH_2_Cl_2_, 40 °C, 0.5 h, 96%; (**e**) Bis(pinacolato)diboron, Pd_2_(dba)_3_, K_2_CO_3_, 85 °C, 6 h, 72%; (**f**) **7**, Pd(PPh_3_)_4_, K_2_CO_3_, 80 °C, 3 h, 77%.

Based on the above hypothesis, a novel series of benzothiazole PI3Kβ inhibitors were designed and synthesized, with the aim of developing compounds with tight interactions with residues ASP856, VAL848, ASP807 and LYS799. The synthetic route of target compound **1** is outlined in Scheme 1.

As shown in Scheme 1, compound **1** and the analogues were prepared by starting from commercially available 2,6-dibromo-4-chloroaniline (**1**). Reaction **a** can occur at room temperature for high yield of 1,3-dibromo-5-chloro-2-isothiocyanatobenzene (**2**). Then, in order to simplify the operation, two reactions of condensation and cyclization were carried out in one step to produce 4-(4-bromo-6-chlorobenzo[d]thiazol-2-yl)morpholine (**3**). Because the reaction activity of bromine atoms is much higher than that of chlorine atom, reaction **c** was prone to occur at the bromine position with almost no 6-position morpholine substitutes. The final step is a typical Suzuki reaction, which could be carried out easily. The over all yield of the whole route is up to 55% [14]. All the compounds synthesized are reported for the first time, and all of the target compounds give satisfactory analytical and spectroscopic data, such as ^1^H-NMR, ^13^C-NMR and HRMS (ESI), which are in accordance with their depicted structures.

### 2.2. Biological Discussion

In order to explore the influences of different substituents to the kinase inhibitory activity of benzothiazole compounds, four substitutes, including hydrogen bond donors or not, were selected to link to C-2 position of benzothiazole, and ethyl substituent were chosen as the R_2_ linked to carbamide group. Compounds **1**–**4** (Table 1) were obtained and then evaluated for inhibitory rates against PI3Kβ kinase at the concentration of 1 μM in vitro together with reference compound GDC-0941 by ADP-Glo Lipid Kinase Assay [15]. The kinase inhibitory rates assay revealed that the activity of compound **1** (52.1%) was significantly higher than that of the compounds **3** (11.7%) and **4** (17.6%) [16]. The result of the first round test confirmed that the compound with the fragment of morpholine in 2-position of benzothiazole (compound **1**) exhibited better inhibitory activity, so the morpholine group was picked as the preferred R_1_ fragment.

After the structure of R_1_ was determined, different substitutes were introduced into the terminal of the carbamide group (Table 2). The enzymatic assay showed that various kinds of terminal structures exhibited significant differences in PI3Kβ inhibitory activity. Among these compounds, compounds **10** and **11** showed the best inhibitory activities against PI3Kβ with the inhibition rate of 75.9% and 88.3% under 1 μM concentration, respectively.

Then, we tested the selectivity of the two compounds for different PI3K Class I subunits and mTOR. The results of kinase assay showed that compounds **10** and **11** had potent activity against PI3Kβ, with excellent selectivity versus PI3Kα, γ, δ and mTOR (Table 3). Different from GDC-0941, compounds **10** and **11** did not possess the widely suppression capabilities on PI3Ks and mTOR, but they revealed obvious selectivity on PI3Kβ. Compared with the inhibitory activity on other kinases, compound **11** displayed the highest potent on inhibition of PI3Kβ (the selectivity is about 208, 289, 154 and 1532 folds against PI3Kα, γ, δ and mTOR, respectively), which suggested our design of compounds was reasonable. The experimental results of PI3K Class I and mTOR enzymatic assay were summarized in Table 3.

The in vitro antitumor activity of compounds **1**–**11** were evaluated in nine tumor cell lines and one human normal cell, of which, PC-3 and DU145 were typically prostate tumor cells. As expected, the results (Table 4) showed compound **11** was superior to compound **10** and GDC-0941 in most of these cell lines, such as A549, MCF7, AGS and SW-620. For the prostate tumor cells PC-3 and DU145, compound **11** showed significantly better performances than compound **10** and GDC-0941, which could be attributed to its excellent PI3Kβ inhibitory activity. Moreover, positive correlation can be found between the anti-prostate tumor activity and PI3Kβ inhibitory activity of compounds **1**–**11**, which could reflect the relationship between anti-prostate tumor activity and PI3Kβ inhibitory activity from a flank. 

Additionally, the most promising compound **11** showed weak inhibitory activity against MRC5 cell line with the IC_50_ values of 33.11 μM (95 and 53 times higher than PC-3 and DU145, respectively), which was significantly weaker than**10** and GDC-0941. The low inhibitory activity of **11** on MRC5 cell reflected its high selectivity against prostate tumor cells, as well as its low cytotoxicity in vitro.

Except for the activity, selectivity and cytotoxicity, the druggability of compound **11** also attracted our attention. The absorption and metabolism property of compound **11** were predicted using software ADMET Predictor version 5.5 (Simulations Plus Inc., Lancaster, CA, USA) [17]. The computer simulation results listed in Table 5 revealed that the absorption and metabolism properties of compound **11** were all in reasonable range, which suggested that **11** was an excellent candidate for potential antitumor agent.

### 2.3. Molecular Docking Study

**Figure 3:**
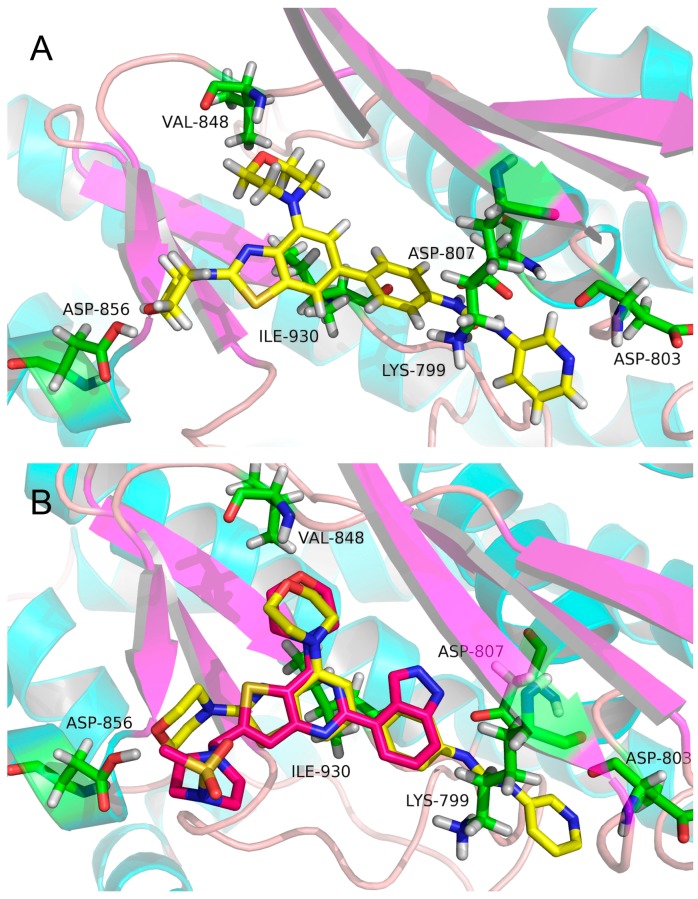
(**A**): schematic of the binding mode of compound **11** with PI3Kβ kinase; (**B**): the contrast of binding mode between compound **11** (yellow) and GDC-0941 (red).

To explore the binding modes of target compounds with the active ATP-binding site of PI3Kβ, molecular docking simulation studies were carried out by using the SURFLEX-DOCK module of the SYBYL 6.9 package version (Tripos International, St. Louis, MO, USA). According to the in vitro kinase inhibition results, we selected compound **11**, the best PI3Kβ inhibitor in this work, as the ligand, and the protein structure of PI3Kβ (PDB ID code: 4BFR) as the docking receptor (Figure 3).

Since numerous studies have suggested that the interaction to the hinge region is crucial to PI3K inhibitory activity [18,19], the interaction with VAL848 is essential to all kinds of PI3K inhibitor. In this model (Figure 3), Compound **11** and GDC-0941 can overlap in the position of morpholine group and both of the morpholine groups bond to the hinge region via the main-chain nitrogen of VAL848. The structure of benzothiazole in **11** is fixed on the central hydrophobic region of the ATP-binding site by π-π conjugation effect with ILE930, the same with the scaffold structure of GDC-0941. ASP807 and LYS799 formed two and one hydrogen bond with the urea group of side chain in **11** from opposite directions, respectively, holding the side chain in the slit of the protein pocket. The pyridine group in the terminal of the side chain interacts with PI3Kβ by an additional hydrogen bond with ASP803 in the C-helix, which can explain why potency of compound **11** is better than compounds **5**–**10**. Given that most residues in the ATP-binding sites of the four PI3K subunits and mTOR are similar, the few different residues play a key role in the selectivity of inhibitors, and the ASP856 is exactly the crucial residue inPI3Kβ. On one hand, ASP856can form a stronger hydrogen bond with compound **11** than LYS890 (PI3Kγ/δ) and ALA2248 (mTOR); on the other hand, the spatial distance between ASP856 and VAL848 is wide enough for structure of 4-(benzo[d]thiazol-2-yl)morpholine of compound **11**. All these factors above contribute to the excellent kinase inhibitory activity and selectivity of compound **11**.

## 3. Materials and Methods 

### 3.1. Chemistry

^1^H-NMR and ^13^C-NMR spectra were recorded at 400 MHz and 100 MHz on a JNM-ECA-400 instrument (JEOL Ltd., Tokyo, Japan) in DMSO-*d*_6_. Chemical shifts are expressed in δ (ppm), with tetramethylsilane (TMS) functioning as the internal reference, coupling constants (J) were expressed in Hz. High-resolution mass spectra were obtained using a TOF G6230A LC/MS (Agilent Technologies, New York, NY, USA) with an ESI source. Melting points were determined using an RY-1 apparatus (Yutong Company, Shanghai, China). Reagents and solvents were commercially available without further purification. The ^1^H-NMR, ^13^C-NMR and HRMS spectra of the compounds in this article can be found in Supplementary Materials.

#### General procedure for the preparation of 1-(4-(2,4-Dimorpholinobenzo[*d*]thiazol-6-yl)phenyl)-3-ethylurea (**1**)

##### Step 1: Preparation of 1,3-Dibromo-5-chloro-2-isothiocyanatobenzene

To a solution of 2,6-dibromo-4-chloroaniline (17.29 g, 60.6 mmol) in methylene chloride (100 mL), thiophosgene (20.9 g, 181.8 mmol) was added quickly at 0 °C. A solution of DMAP in methylene chloride (100 mL) was added into the reaction mixture, and the mixture was stirred at 20 °C for 5 h [20]. The reaction was quenched with distilled water, and the organic phase was washed with water and brine, dried over Na_2_SO_4_, filtered and concentrated in vacuo. The resultant residue was purified by column chromatography to get the intermediate as a white solid (18.86 g, 57.6 mmol, 95% yield). ^1^H-NMR (400 MHz, DMSO-*d*_6_) δ (ppm): 8.00 (s, 2H, Ar-H). MS *m*/*z* [M + H]^+^: 325.8.

##### Step 2: Preparation of 4-(4-Bromo-6-chlorobenzo[*d*]thiazol-2-yl)morpholine

A mixture of 1,3-dibromo-5-chloro-2-isothiocyanatobenzene(18.86 g, 57.6 mmol) and morpholine (5.27 g, 60.48 mmol) was dissolved into dioxane (250 mL) and stirred at 25 °C for 30 min. Then, caesium carbonate (28.15 g, 86.4 mmol) was added into the reaction mixture, and the mixture was stirred at 105 °C for 3 h [21]. The reaction was cooled to 20 °C and quenched with distilled water. The aqueous phase was extracted with dichloromethane, and the combined organic phase was washed with water and brine, dried over Na_2_SO_4_, filtered and concentrated in vacuo. The resultant residue was purified by column chromatography to get the intermediate as a white solid (17.68 g, 52.99 mmol, 92% yield). ^1^H-NMR (400 MHz, CDCl_3_) δ (ppm): 7.51 (d, *J* = 2.0 Hz, 1H, Ar-H), 7.49 (d, *J* = 2.0 Hz, 1H, Ar-H), 3.86–3.81 (m, 4H, OCH_2_), 3.67–3.62 (m, 4H, NCH_2_). MS *m*/*z* [M + H]^+^: 332.9.

##### Step 3: Preparation of 4,4′-(6-Chlorobenzo[*d*]thiazole-2,4-diyl)dimorpholine.

A mixture of 4-(4-bromo-6-chlorobenzo[d]thiazol-2-yl)morpholine (17.68 g, 52.99 mmol), morpholine (5.54 g, 63.59mmol), Tris(dibenzylideneacetone)dipalladium(0) (3.4 g, 3.71 mmol), *R*-BINAP (2.31 g, 3.71 mmol), and caesium carbonate (25.89 g, 79.49 mmol) was dissolved into DMF (200 mL) and stirred at 90 °C for 8 h under a nitrogen atmosphere. The reaction was cooled to 20 °C and quenched with distilled water. The aqueous phase was extracted with dichloromethane, and the combined organic phase was washed with water and brine, dried over Na_2_SO_4_, filtered and concentrated in vacuo. The resultant residue was purified by column chromatography to get the intermediate as a white solid (14.77 g, 43.45 mmol, 82% yield). ^1^H-NMR (400 MHz, DMSO-*d*_6_) δ (ppm): 7.38 (s, 1H, Ar-H), 6.62 (s, 1H, Ar-H), 3.75–3.72 (m, 4H, OCH_2_), 3.52–3.50 (m, 4H, OCH_2_), 2.99 (s, 8H, NCH_2_). MS *m*/*z* [M + H]^+^: 340.1.

##### Step 4: Preparation of 1-(4-(2,4-Dimorpholinobenzo[*d*]thiazol-6-yl)phenyl)-3-ethylurea

A mixture of 4,4′-(6-chlorobenzo[*d*]thiazole-2,4-diyl)dimorpholine (14.77 g, 43.45 mmol), [(4-Ethylureido)phenyl] boronic acid pinacol ester (18.91 g, 65.18 mmol), tetrakis(triphenylphosphine)palladium(0) (3.51 g, 3.04 mmol), and potassium carbonate (18.02 g, 130.38 mmol) was dissolved into a mixture solution of glycol dimethyl ether (300 mL) and distilled water (60 mL). Then, the mixture was stirred at 80 °C for 3 h under a nitrogen atmosphere [22]. Reaction was cooled to 20 °C and quenched with distilled water. The aqueous phase was extracted with dichloromethane, and the combined organic phase was washed with water and brine, dried over Na_2_SO_4_, filtered and concentrated in vacuo. The resultant residue was purified by column chromatography to get the intermediate as a white solid (15.64 g, 33.46 mmol, 77% yield). m.p.: 256–260 °C. ^1^H-NMR (400 MHz, DMSO-*d*_6_) δ (ppm): 8.51 (s, 1H, NH),7.63 (s, 1H, Ar-H), 7.55 (d, *J* = 8.7 Hz, 2H, Ar-H), 7.45 (d, *J* = 8.7 Hz, 2H, Ar-H), 6.95 (s,1H, Ar-H), 6.11 (t, *J* = 5.5 Hz, 1H, NH), 3.78 (dt, *J* = 21.4, 4.6 Hz, 8H, OCH_2_), 3.58–3.47 (m, 8H, NCH_2_), 3.19–3.04 (m, 2H, CH_2_), 1.06 (t, *J* = 7.2 Hz, 3H, CH_3_).^13^C-NMR (100 MHz, DMSO-*d*_6_) δ (ppm): 166.2 (C), 155.0 (CO), 142.8(C), 142.2 (C), 139.7 (C), 134.4 (C), 133.1 (C), 132.2 (C), 126.7 (CH), 117.9 (CH), 111.1 (CH), 111.0 (CH), 66.3 (CH_2_), 65.5 (CH_2_), 50.0 (CH_2_), 48.0 (CH_2_), 33.9 (CH_2_), 15.5 (CH_3_). HRMS (ESI+) *m*/*z* [M + H]^+^ calculated for C_24_H_29_N_5_O_3_S: 467.20; found: 468.2066.

*Ethyl 4-(6-(4-(3-ethylureido)phenyl)-4-morpholinobenzo[d]thiazol-2-yl) piperazine-1-carboxylate* (**2**) (total yield: 27.4%). m.p.: 248–251 °C. ^1^H-NMR (400 MHz, DMSO-*d*_6_) δ (ppm): 8.51 (s, 1H, NH), 7.63 (s, 1H, NH), 7.54 (d, *J* = 8.8 Hz, 2H, Ar-H), 7.45 (d, *J* = 8.8 Hz, 2H, Ar-H), 6.94 (s, 1H, Ar-H), 6.11 (t, *J* = 5.6 Hz, 1H, Ar-H), 4.08 (q, *J* = 7.1 Hz, 2H, OCH_2_), 3.84–3.78 (m, 4H, OCH_2_), 3.55 (s, 8H, NCH_2_), 3.36 (d, *J* = 4.2 Hz, 4H, NCH_2_), 3.11 (td, *J* = 7.2,5.6 Hz, 2H, NCH_2_), 1.21 (t, *J* = 7.1 Hz, 3H, CH_3_), 1.06 (t, *J* = 7.2 Hz, 3H, CH_3_). ^13^C-NMR (100 MHz, DMSO-*d*_6_) δ (ppm): 166.4 (C), 155.6 (CO_2_), 155.1 (CO), 143.3 (C), 142.8 (C), 140.2 (C), 134.9 (C), 133.6 (C), 132.9 (C), 127.3 (CH), 118.4 (CH), 111.7 (CH), 111.6 (CH), 66.8 (CH_2_), 61.6 (CH_2_), 50.6 (CH_2_), 48.2 (CH_2_), 43.2 (CH_2_), 34.5 (CH_2_), 16.1 (CH_3_), 15.1 (CH_3_). HRMS (ESI+) *m*/*z* [M + H]^+^ calculated for C_27_H_34_N_6_O_4_S: 538.24; found: 539.2437. 

*1-Ethyl-3-(4-(2-(4-isopropylpiperazin-1-yl)-4-morpholinobenzo[d]thiazol-6-yl)phenyl)urea* (**3**) (total yield: 24.5%). m.p.: 200–202 °C. ^1^H-NMR (400 MHz, DMSO-*d*_6_) δ (ppm): 8.51 (s, 1H, NH), 8.03 (s, 1H, NH), 7.54 (d, *J* = 8.7 Hz, 2H, Ar-H), 7.45 (d, *J* = 8.7 Hz, 2H, Ar-H), 6.94 (s, 1H, Ar-H), 6.11 (t, *J* = 5.6 Hz, 1H, Ar-H), 3.85–3.76 (m, 4H, OCH_2_), 3.53 (q, *J* = 7.4, 4.7 Hz, 2H, CH_2_), 3.18–3.05 (m, 8H, NCH_2_), 2.79–2.68 (m, 1H, NCH), 2.59–2.55 (m, 4H, NCH_2_), 1.06 (t, *J* = 7.2 Hz, 3H, CH_3_),1.00 (d, *J* = 6.5 Hz, 6H, CH_3_). ^13^C-NMR (100 MHz, DMSO-*d*_6_) δ (ppm), 166.4 (C), 155.60 (CO), 151.9 (C), 143.2 (C), 140.20 (C), 133.9 (C), 132.8 (C), 131.9 (C), 127.3 (CH), 124.7 (CH), 118.5 (CH), 111.7 (CH), 66.8 (CH_2_), 54.4 (CH), 50.6 (CH_2_), 48.9 (CH_2_), 47.9 (CH_2_), 34.5 (CH_2_), 18.6 (CH_3_), 16.0 (CH_3_). HRMS (ESI+) *m*/*z* [M + H]^+^ calculated for C_27_H_36_N_6_O_2_S: 508.26; found: 509.2694. 

*1-Ethyl-3-(4-(4-morpholino-2-(piperidin-1-yl)benzo[d]thiazol-6-yl)phenyl)urea* (**4**) (total yield: 17.2%). m.p.: 228–231 °C. ^1^H-NMR (400 MHz, DMSO-*d*_6_) δ (ppm): 8.50 (s, 1H, NH), 7.59 (s, 1H, NH), 7.53 (d, *J* = 8.8 Hz, 2H, Ar-H), 7.45 (d, *J* = 8.8 Hz, 2H, Ar-H), 6.93 (s, 1H, Ar-H), 6.11 (t, *J* = 5.6 Hz, 1H, Ar-H), 3.87–3.76 (m, 4H, OCH_2_), 3.42–3.32 (m, 4H, NCH_2_), 3.42–3.32 (m, 2H, CH_2_), 3.17–3.05 (m, 4H, NCH_2_), 1.66–1.59 (m, 4H, CH_2_), 1.58–1.56 (m, 2H, CH_2_), 1.06 (t, *J* = 7.2 Hz, 3H, CH_3_). ^13^C-NMR (100 MHz, DMSO-*d*_6_) δ (ppm): 166.4 (C), 155.6 (CO), 143.2 (C), 142.9 (C), 140.1 (C), 134.5 (C), 133.7 (C), 132.7 (C), 127.2 (CH), 118.4 (CH), 111.6 (CH), 111.5 (CH), 66.8 (CH_2_), 50.6 (CH_2_), 49.5 (CH_2_), 34.5 (CH_2_), 25.4 (CH_2_), 24.2 (CH_2_), 16.0 (CH_3_). HRMS (ESI+) *m*/*z* [M + H]^+^ calculated for C_25_H_31_N_5_O_2_S: 465.22; found: 466.2271.

*1-Cyclopropyl-3-(4-(2,4-dimorpholinobenzo[d]thiazol-6-yl)phenyl)urea* (**5**) (total yield: 27.2%). m.p.: 238–240 °C. ^1^H-NMR (400 MHz, DMSO-*d*_6_) δ (ppm): 8.39 (s, 1H, NH), 7.64 (s, 1H, Ar-H), 7.55 (d, *J* = 8.6 Hz, 2H, Ar-H), 7.47 (d, *J* = 8.4 Hz, 2H, Ar-H), 6.96 (s, 1H, Ar-H), 6.41 (s, 1H, NH), 3.76 (dt, *J* = 21.1, 4.3 Hz, 8H, OCH_2_), 3.58–3.46 (m, 8H, NCH_2_), 2.58–2.47 (m, 1H, CH), 0.64 (q, *J* = 6.1, 5.4 Hz, 2H, CH_2_), 0.42 (q, *J* = 6.1, 5.4 Hz, 2H, CH_2_). ^13^C-NMR (100 MHz, DMSO-*d*_6_) δ (ppm): 166.3(C), 155.9 (CO), 142.8 (C), 142.3 (C), 139.4 (C), 134.4 (C), 133.3 (C), 132.2 (CH), 126.7 (CH), 118.1 (CH), 111.2 (CH), 66.3 (CH_2_), 65.5 (CH_2_), 50.1 (CH_2_), 48.1 (CH_2_), 22.4 (CH), 6.5 (CH_2_). HRMS (ESI+) *m*/*z* [M + H]^+^ calculated for C_25_H_29_N_5_O_3_S: 479.20; found: 480.2064.

*1-Benzyl-3-(4-(2,4-dimorpholinobenzo[d]thiazol-6-yl)phenyl)urea* (**6**) (total yield: 24.5%). m.p.: 252–255 °C. ^1^H-NMR (400 MHz, DMSO-*d*_6_) δ (ppm): 8.67 (s, 1H, NH), 8.61 (s, 1H, NH), 7.64 (s, 1H, Ar-H), 7.55 (d, *J* = 8.0 Hz, 2H, Ar-H), 7.49 (d, *J* = 8.0 Hz, 2H, Ar-H), 7.33 (d, *J* = 8.0 Hz, 2H, Ar-H), 7.32 (d, *J* = 8.0 Hz, 2H, Ar-H), 6.95 (s, 1H, Ar-H), 6.67–6.63 (m, 1H, Ar-H), 4.32 (d, *J* = 8.0 Hz, 2H, CH_2_), 3.82–3.78 (m, 4H, CH_2_), 3.77–3.73 (m, 4H, CH_2_), 3.56–3.51 (m, 4H, CH_2_), 3.40–3.34 (m, 4H, CH_2_). ^13^C-NMR (100 MHz, DMSO-*d*_6_) δ (ppm): 166.3 (C), 155.2 (CO), 142.8 (C),142.3 (C), 140.4 (C), 139.5 (C), 134.3 (C), 133.2 (C), 132.2 (CH), 128.3 (CH), 127.1 (CH), 126.8 (CH), 126.7 (CH), 117.9 (CH), 111.2 (CH), 66.3 (CH_2_), 65.5 (CH_2_), 50.0 (CH_2_), 48.0 (CH_2_), 42.9 (CH_2_). HRMS (ESI+) *m*/*z* [M + H]^+^ calculated for C_29_H_31_N_5_O_3_S: 529.21; found: 530.2221.

*1-(2,4-Difluorophenyl)-3-(4-(2,4-dimorpholinobenzo[d]thiazol-6-yl)phenyl)urea* (**7**) (total yield: 31.5%). m.p.: 244–247 °C. ^1^H-NMR (400 MHz, DMSO-*d*_6_) δ (ppm): 9.22 (s, 1H, NH), 8.61(s, 1H, NH), 8.10 (s, 1H, Ar-H), 7.67 (s, 1H, Ar-H), 7.63 (d, *J* = 7.9 Hz, 2H, Ar-H), 7.53 (d, *J* = 7.9 Hz, 2H, Ar-H), 7.37–7.29 (m, 1H, Ar-H), 7.10–7.03 (m, 1H, Ar-H), 6.98 (s, 1H, Ar-H), 3.80 (d, *J* = 4.0 Hz, 8H, OCH_2_), 3.58–3.49 (m, 4H, NCH_2_), 3.37 (s, 4H, NCH_2_). ^13^C-NMR (100 MHz, DMSO-*d*_6_) δ (ppm): 166.4 (C), 152.4 (CO), 142.8 (C), 142.4 (C), 138.6 (C), 134.2 (C), 132.3 (C), 126.9 (C), 124.2 (CH), 122.2 (CH), 122.1 (CH), 118.4 (CH), 111.4 (CH), 111.2 (CH), 103.8 (CH), 66.3 (CH_2_), 65.5 (CH_2_), 50.1 (CH_2_), 48.1 (CH_2_). HRMS (ESI+) *m*/*z* [M + H]^+^ calculated for C_28_H_27_F_2_N_5_O_3_S: 551.18; found: 552.1875.

*1-(4-(2,4-Dimorpholinobenzo[d]thiazol-6-yl)phenyl)-3-methylurea* (**8**) (total yield: 20.3%). m.p.: 274–278 °C. ^1^H-NMR (400 MHz, DMSO-*d*_6_) δ (ppm): 8.59 (s, 1H, NH), 7.63 (s, 1H, Ar-H), 7.55 (d, *J* = 8.8 Hz, 2H, Ar-H), 7.46 (d, *J* = 8.80 Hz, 2H, Ar-H), 6.95 (s, 1H, Ar-H), 6.02 (s, 1H, NH),3.77 (dt, *J* = 21.2, 4.7 Hz, 8H, OCH_2_), 3.55–3.50 (m, 4H, NCH_2_), 3.35 (d, *J* = 7.5 Hz, 4H, NCH_2_), 2.65 (d, *J* = 4.6 Hz, 3H, CH_3_). ^13^C-NMR (100 MHz, DMSO-*d*_6_) δ (ppm): 166.8 (C), 156.3 (CO), 143.3 (C), 142.8 (C), 140.3 (C), 134.9 (C), 133.6 (C), 132.7 (CH), 127.3 (CH), 118.4 (CH), 111.7 (CH), 66.8 (CH_2_), 66.0 (CH_2_), 50.6 (CH_2_), 48.6 (CH_2_), 26.8 (CH_3_). HRMS (ESI+) *m*/*z* [M + H]^+^ calculated for C_23_H_27_N_5_O_3_S: 453.18; found: 454.1908.

*1-(4-(2,4-Dimorpholinobenzo[d]thiazol-6-yl)phenyl)-3-(4-(4-methylpiperazin-1-yl)phenyl)urea* (**9**) (total yield: 17.6%). m.p.: 214–216 °C. ^1^H-NMR (100 MHz, DMSO-*d*_6_) δ (ppm): 7.95 (s, 1H, NH), 7.65 (s, 1H, Ar-H), 7.56 (d, *J* = 8.9 Hz, 2H, Ar-H), 7.52 (d, *J* = 8.9 Hz, 2H, Ar-H), 7.35 (d, *J* = 9.0 Hz, 2H, Ar-H), 7.10 (s, 1H, Ar-H), 6.97 (s, 1H, NH), 6.86 (d, *J* = 9.0 Hz, 2H, Ar-H), 3.78 (dt, *J* = 21.2,4.1 Hz, 4H, OCH_2_), 3.56–3.50 (m, 2H, OCH_2_), 3.07–3.01 (m, 2H, OCH_2_), 3.06–3.01 (m, 4H, CH_2_), 2.93–2.96 (m, 4H, NCH_2_), 2.73 (s, 12H, NCH_2_), 2.22 (s, 3H, CH_3_). ^13^C-NMR (400 MHz, DMSO-*d*_6_) δ (ppm): 165.9 (C), 154.6 (CO), 152.7 (CO), 146.5 (C), 142.8 (C), 142.4 (C), 139.0 (C), 134.3 (C), 132.4 (C), 131.6 (C), 126.9 (C), 119.7 (CH), 118.3 (CH), 116.1 (CH), 111.3 (CH), 95.4 (CH), 66.3 (CH), 66.3 (CH_2_), 65.2 (CH_2_), 61.0 (CH_2_), 54.7 (CH_2_), 50.1 (CH_2_), 48.8 (CH_2_), 47.6 (CH_3_), 45.8 (CH_2_), 14.6 (CH_3_). HRMS (ESI+) *m*/*z* [M + H]^+^ calculated for C_33_H_39_N_7_O_3_S: 613.28; found: 614.2906.

*1-(4-(2,4-Dimorpholinobenzo[d]thiazol-6-yl)phenyl)-3-(4-(hydroxymethyl)phenyl)urea* (**10**) (total yield: 22.7%). m.p.: 223–224°C. ^1^H-NMR (400 MHz, DMSO-*d*_6_) δ (ppm): 8.73 (s, 1H, NH), 8.64 (s, 1H, NH), 7.66 (d, *J* = 1.6 Hz, 1H, Ar-H), 7.61 (d, *J* = 8.8 Hz, 2H, Ar-H), 7.52 (d, *J* = 8.8 Hz, 2H, Ar-H), 7.41 (d, *J* = 8.5 Hz, 2H, Ar-H), 7.23 (d, *J* = 8.5 Hz, 2H, Ar-H), 6.97 (d, *J* = 1.7 Hz, 1H, Ar-H), 5.06 (t, *J* = 5.7 Hz, 1H, OH), 4.43 (d, *J* = 5.7 Hz, 2H, CH_2_), 3.78 (dt , *J* = 22.0,4.7 Hz, 8H, OCH_2_), 3.57–3.50 (m, 8H, NCH_2_). ^13^C-NMR (100 MHz, DMSO-*d*_6_) δ (ppm): 166.3 (C), 152.5 (CO), 142.8 (C), 142.4 (C), 138.8 (C), 138.3 (C), 135.9 (C), 134.3 (C), 133.9 (C), 132.2 (CH), 127.2 (CH), 126.9 (CH), 126.8 (CH), 118.4 (CH), 117.9 (CH), 66.3 (CH_2_), 65.5 (CH_2_), 62.7 (CH_2_), 50.0 (CH_2_), 48.0 (CH_2_). HRMS (ESI+) *m*/*z* [M + H]^+^ calculated for C_29_H_31_N_5_O_4_S: 545.21; found: 546.2171.

*1-(4-(2,4-Dimorpholinobenzo[d]thiazol-6-yl)phenyl)-3-(pyridin-3-yl)urea* **(11)** (total yield: 16.2%). m.p.: 209–211 °C. ^1^H-NMR (400 MHz, DMSO-*d*_6_) δ (ppm): 8.90 (s, 1H, Ar-H), 8.87 (s, 1H, Ar-H), 8.6 2 (d, *J* = 2.0 Hz, 1H, Ar-H), 8.20 (d, *J* = 5.6 Hz, 1H, Ar-H), 7.96 (d, *J* = 10.2 Hz, 1H, Ar-H), 7.66 (s, 1H, Ar-H), 7.62 (d, *J* = 8.7 Hz, 2H, Ar-H), 7.53 (d, *J* = 8.7 Hz, 2H, Ar-H), 7.33 (dd, *J* = 8.2, 4.5 Hz, 1H, Ar-H), 6.98 (d, *J* = 1.5 Hz, 1H, Ar-H), 3.78 (dt, *J* = 22.2, 4.4 Hz, 8H, OCH_2_), 3.56–3.51 (m, 8H, NCH_2_). ^13^C-NMR (100 MHz, DMSO-*d*_6_) δ (ppm): 166.8 (C), 153.1 (CO), 143.3 (C), 142.9 (C), 140.6 (C), 138.9 (C), 134.8 (C), 134.7 (C), 132.7 (CH), 127.5 (CH), 125.7 (CH), 124.2 (CH), 119.2 (CH), 118.9 (CH), 117.7 (CH), 95.4 (CH), 66.8 (CH_2_), 66.0 (CH_2_), 50.6 (CH_2_), 48.6 (CH_2_). HRMS (ESI+) *m*/*z* [M + H]^+^ calculated for C_27_H_28_N_6_OS: 516.19; found: 517.2017.

### 3.2. Pharmacology

#### 3.2.1. Class I PI3Ks Enzyme Assay

The inhibition of PI3Ks (PI3Kα, PI3Kβ, PI3Kγ, PI3Kδ) activity was determined using ADP-Glo kinase assay (PI3Kα, PI3Kβ, PI3Kγ and PI3Kδ were all purchased from Promega Corporation (Madison, Wisconsin, USA, #V1691), respectively. Before the assay, some reagent should be prepared as the protocol offered by Promega. Ten microliters (1 μg, Promega #V1691) of PI3K enzyme were diluted in the 310 μL 2.5× kinase reaction buffer (Promega, #V1691) to give 2.5× kinase solutions. Fifty microliters PIP2:3PS substrate and ATP were diluted in the 100 µL 10× lipid dilution buffer and 250 µL water to give 2.5× PIP2:3PS lipid kinase substrate working solution. Twenty-five microliters ultra pure ATP (10 mM) was diluted in the 975 µl water to get 250 µM ATP water solution. 

When the experiment began, test compounds were serially diluted to the desired concentrations and then 1 μL of each of them was added to a 384-well plate (Corning, New York, NY, USA) as assay plate [23]. Four microliters of PIP2:3PS lipid kinase substrate working solution was added to each well of the assay plate. Then, 4 μL of kinase solution was added to each well of the assay plate, except for control well add 4μL of 1x kinase reaction buffer instead (on enzyme control group). The kinase reaction started by adding 1 μL of 250 μM ATP, and then the assay plate was covered, mixed for 30 to 60 s, and incubated 1 h at 23°C (room temperature). Ten microliters of ADP-Glo™ reagent (Promega) was added into the reaction mixture to stop the enzyme reaction and depleted unconsumed ATP. Forty minutes later, 20 μL kinase detection reagent (Promega) were added into the reaction mixture to convert ADP to ATP. The mixture was shaken for 1 minute and equilibrated for 40 minutes before reading on a plate reader for luminescence. Finally, conversion data was collected on Flex station and RLU (relative light unit) values were converted to inhibition values using the formula of (max − Sample RLU)/(max − min) × 100%. Herein, “max” means the RLU of DMSO control and “min” means the RLU of no enzyme control [20], All of the compounds were tested two times, and the results expressed as IC_50_ (inhibitory concentration 50%) were the averages of two determinations.

#### 3.2.2. mTOR Enzyme Assay

The mTOR kinase activities of all the compounds were determined using LANCE^®^ ultra time-resolved fluorescence resonance energy transfer (TR-FRET) assay (Invitrogen, Carlsbad, CA, USA) [20] following the manufacturer’s instructions, with compound GDC-0941 as positive controls. Briefly, mTOR enzyme (0.1 μg/mL, Invitrogen), ATP (3 μM), GFP-4EBP1 Peptide (0.4 μM) and test compounds were diluted in kinase buffer (50 mM HEPES pH 7.5, 1 mM EGTA, 3 mM MnCl_2_, 10 mM MgCl_2_, 2 mM DTT and 0.01% Tween-20). The reactions were performed in black 384-well proxiplates (Corning) at room temperature for 1 h and stopped by adding EDTA to 10 mM. Tb-antiphospho-4EBP1 (Thr37/46) Antibody (PerkinElmer, Fremont, CA, USA) was then added to each well to a final concentration of 2 nM, and the mixture was incubated at room temperature for 30 min. The intensity of the light emission was measured with Spectramax 190 reader (Molecular Devices, Valley, CA, USA) in TR-FRET mode (excitation at 320 nm and emission at 665 nm). All of the compounds were tested two times, and the results expressed as IC_50_ (inhibitory concentration 50%) were the averages of two determinations.

#### 3.2.3. Anti-Proliferative Assay

All new synthesized compounds in this paper were screened for their anti-proliferative activity by MTS assay. A549, MCF7, SKOV3, AGS, MES-SA, HepG2, SW-620, PC-3, DU145 and MRC5 cells in this paper were all purchased from American Type Culture Collection (ATCC, Manassas, VA, USA), and were seeded into 96-well plates and cultured for 24 h. Subsequently, these cells were exposed to serial concentrations of compound for 72 h. Cells were then washed with PBS and fixed with CellTiter 96^®^ (Promega) aqueous one solution reagent at 37°C for 3 h. Finally, record the absorbance at 490 nm using a 96-well plate reader. The inhibition rate of cell proliferation of each well was calculated using the formula of (control cells − treated cells)/control cells × 100%. All of the compounds were tested two times in each of the cell lines and the results expressed as inhibition rates or IC_50_ (inhibitory concentration 50%) were the averages of two determinations.

### 3.3. Molecular Docking Study

Molecular docking process: first of all, crystal structures of human PI3K subunits and mTOR were downloaded on to the RCSB Protein Data Bank (http://www.rcsb.org). The selected protein Data Bank (PDB) ID was: human PI3Kα (3ZIM, resolution 2.85 Å), PI3Kβ (4BFR, resolution 2.8 Å), PI3Kγ (5EDS, resolution 2.8 Å) and PI3Kδ (4GB9, resolution 2.44 Å). Next, the water molecules in the crystal were cleared, and hydrogen atoms and electric charges were added. Subsequently, molecule structures of ligands were drawn by ChemBioDraw 12.0 (PerkinElmer), and introduced into protein crystal cells. Finally, the molecular docking was carried out by using the SURFLEX-DOCK module of SYBYL 6.9 package version (Tripos International, St. Louis, MO, USA). After the docking process was over, the best conformation (Figure 3) was selected and the hydrogen bonds were displayed according to the docking result.

## 4. Conclusions

In summary, by comparing and analyzing these PI3K subunits, we selected a unique residue ASP856 in PI3Kβ, which could help us design a highly selective inhibitor. Subsequently, a series of benzothiazole derivatives were developed. All of the compounds were evaluated for kinase (PI3Ka, β, γ, δ and mTOR) inhibitory activity and anti-proliferative (A549, MCF7, SKOV3, AGS, MES-SA, HepG2, SW-620, PC-3, DU145 and MRC5 cell lines) activity in vitro. The pharmacological results indicated that compound **11** has significant inhibition ability and selectivity on PI3Kβ (PI3Kβ IC_50_ = 0.02 μM) and prostate tumor cells (PC-3 IC_50_ = 0.35 μM, DU145 IC_50_ = 0.62 μM) in vitro. Molecular modeling suggests that morpholine group on the 2-position of benzothiazole in compound **11** forms an essential hydrogen bond with ASP856 in PI3Kβ, thus leading to high selectivity of compound **11**. In conclusion, compound **11** has quite good preliminary performance for activity, selectivity, cytotoxicity and druggability, which suggested that compound **11** was an excellent candidate for the PI3Kβ inhibitor. Research on these compounds is ongoing, and further efforts are in progress to synthesize more structurally diverse analogues and evaluate anti-prostate cancer activity in vivo.

## Figures and Tables

**Table 1:**
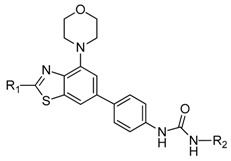
Structures and inhibition rates of compounds **1**–**4** against PI3Kβ kinase at 1 μM.

Compd.	R_1_	R_2_	% Inhib. 1 μM
**1**	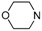	ethyl	52.1%
**2**	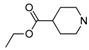	ethyl	23.0%
**3**	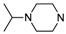	ethyl	11.7%
**4**	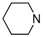	ethyl	17.6%
GDC-0941	/	/	74.5%

**Table 2:**
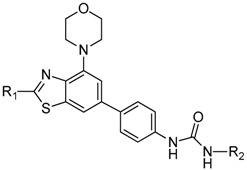
Structures and inhibition rates of compounds **5**–**11** against PI3Kβ kinase at 1 μM.

Compd.	R_1_	R_2_	PI3Kβ, 1 μM, %
**1**	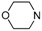	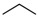	52.1%
**5**	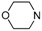	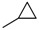	10.2%
**6**	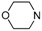	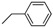	15.0%
**7**	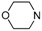	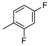	37.7%
**8**	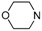	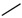	27.2%
**9**	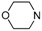	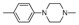	22.5%
**10**	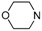	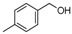	75.9%
**11**	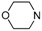	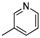	88.3%
GDC-0941	/	/	74.5%

PI3Kβ: Phosphatidylinositol-3-kinase beta subunit.

**Table 3:** Enzymatic activities of compounds **10** and **11** against PI3Kα, β, γ, δ and mTOR (IC_50_, μM).

Compd.	IC_50_, μM				
	PI3Kβ	PI3Kα	PI3Kγ	PI3Kδ	mTOR
**10**	0.12	>50	>50	15.31	>50
**11**	0.02	5.43	7.52	4.01	39.85
GDC-0941	0.15	0.01	0.27	0.01	1.70

PI3Kα: Phosphatidylinositol-3-kinase alpha subunit; PI3Kβ: Phosphatidylinositol-3-kinase beta subunit; PI3Kγ: Phosphatidylinositol-3-kinase gamma subunit; PI3Kδ: Phosphatidylinositol-3-kinase delta subunit; mTOR: Mammalian target of rapamycin.

**Table 4:** Anti-proliferative activities of compounds **1**–**11** and GDC-0941.

Compd.	IC_50_, μM									
	A549	MCF7	SKOV3	AGS	MES-SA	HepG2	SW-620	PC-3	DU145	MRC5
**1**	>100	4.06	21.23	9.69	12.31	>100	8.31	15.32	9.50	6.06
**2**	>100	4.04	>100	>100	>100	>100	>100	85.00	>100	>100
**3**	2.27	6.53	3.76	15.35	3.92	6.20	10.93	25.23	71.69	3.53
**4**	14.77	6.58	5.59	12.16	21.85	>100	>100	33.50	>100	83.47
**5**	14.39	40.78	26.86	64.94	33.15	>100	30.78	>100	>100	38.50
**6**	5.67	19.94	8.85	46.97	24.90	>100	41.25	>100	>100	16.52
**7**	3.72	14.17	18.92	36.60	6.49	15.83	>100	14.17	22.98	28.26
**8**	7.53	11.55	20.43	>100	>100	9.83	>100	25.19	17.53	35.69
**9**	32.69	>100	35.20	37.92	75.29	>100	82.65	42.40	38.55	>100
**10**	84.96	0.75	0.63	1.78	0.72	2.70	5.39	5.74	2.95	1.19
**11**	3.48	0.36	2.64	0.40	0.55	3.43	1.17	0.35	0.62	33.11
GDC-0941	6.91	0.89	0.30	3.62	0.23	1.10	3.27	5.92	1.41	20.72

**Table 5:** Absorption and metabolism property of compound **11**.

Compd.	Property of Absorption and Metabolism		
	MlogP ^a^	Absn Risk ^b^	CYP Risk ^c^
**11**	1.8	2.8	1.5

^a^ Oil-water partition coefficient, suggested values: MlogP < 4.15. ^b^ Druggability risk about absorption, suggested values: Absn Risk ≤ 3. ^c^ Druggability risk about metabolism, suggested values: CYP Risk ≤ 2.

## Supplementary Materials

Supplementary materials can be accessed at: http://www.mdpi.com/1420-3049/21/7/876/s1.

## Abbreviations

The following abbreviations are used in this manuscript:

PI3K: Phosphatidylinositol-3-kinases
mTOR: Mammalian target of rapamycin
PTEN: Phosphatase and tensin homolog deleted on chromosome ten
DNA-PK: DNA-dependent protein kinase
ATM: Ataxia-telangiectasia mutated
ATR: ATM- and Rad3-related
R-BINAP: (*R*)-(+)-2,2′-Bis(diphenylphosphino)-1,1′-binaphthalene
HEPES: 4-(2-Hydroxyethyl)-1-piperazineethanesulfonic acid
EGTA: Ethylene glycol tetraacetic acid
EDTA: Ethylene diamine tetraacetic acid
DTT: Dithiothreitol
TR-FRET: Time-resolved fluorescence resonance energy transfer
RLU: relative light unit
