# Supplementary material for: Design, Synthesis and Biological Evaluation of Novel Benzothiazole Derivatives as Selective PI3Kβ Inhibitors
Source: Molecules. 2016 Jul 2;21(7):876. doi: 10.3390/molecules21070876 (PMC6274018; doi:10.3390/molecules21070876)
Supplement: Supplementary file 1 [file molecules-21-00876-s001.pdf]

Supplementary Materials: Design, Synthesis and Biological Evaluation of Novel Benzothiazole Derivatives as Selective PI3Kβ Inhibitors

Shuang Cao, Ruiyuan Cao, Xialing Liu, Xiang Luo and Wu Zhong

Content

^{1}H-NMR, ^{13}C-NMR and HRMS spectra of compound **1–11**.

1-(4-(2,4-Dimorpholinobenzo[d]thiazol-6-yl)phenyl)-3-ethylurea (**1**)

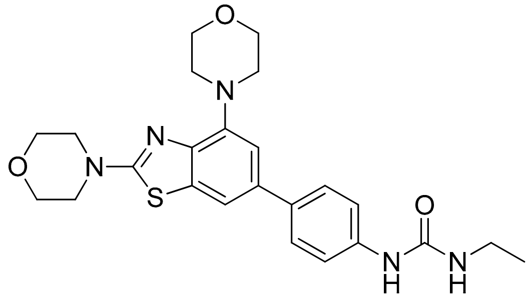

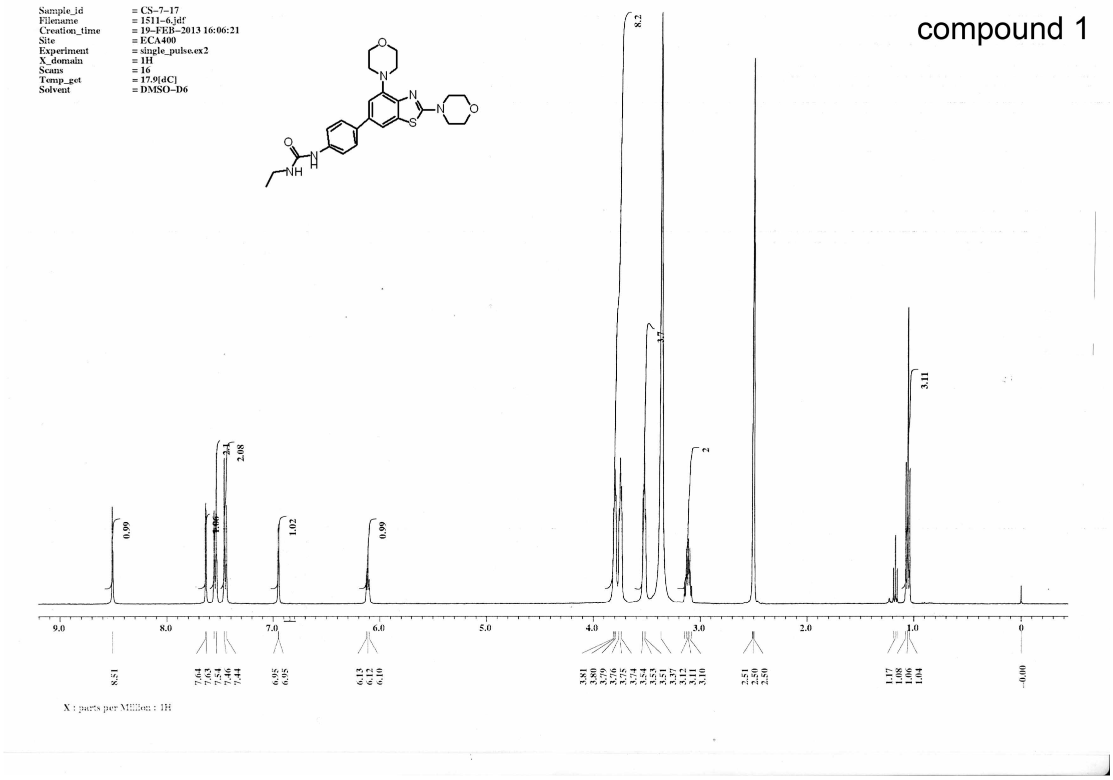

Figure S1. ^{1}H-NMR of compound **1**.

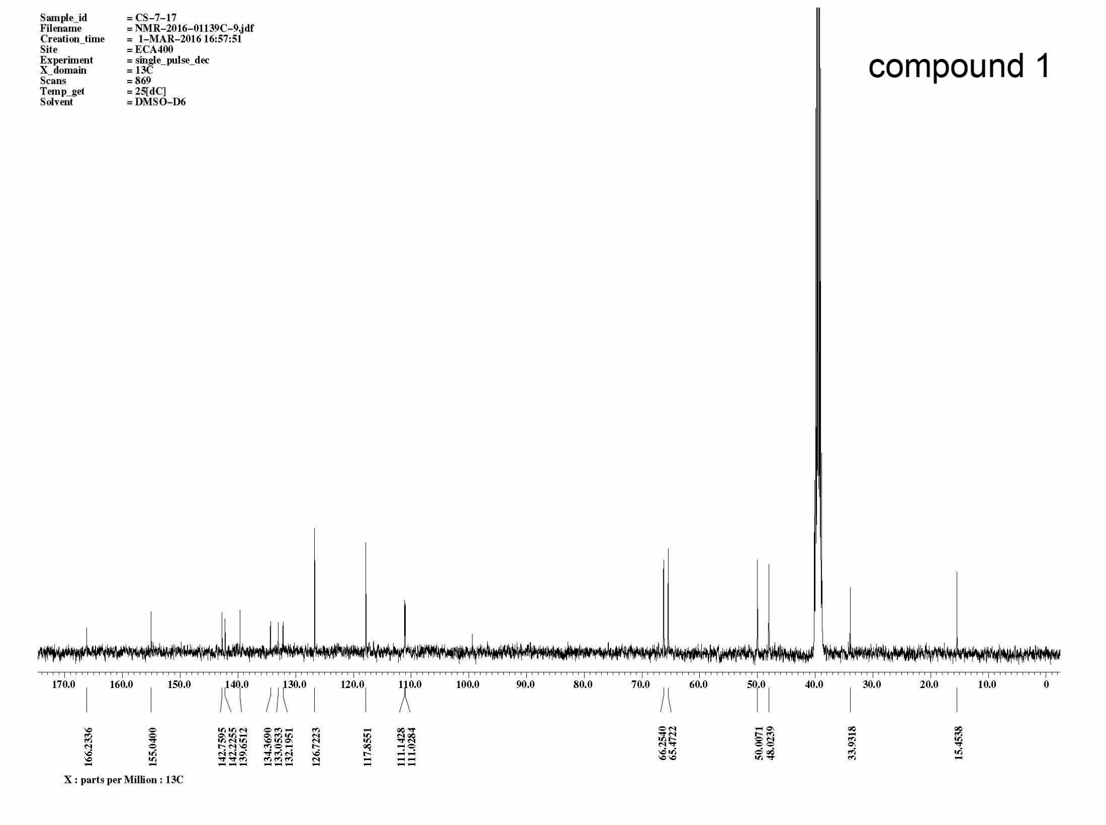

Figure S2. ^{13}C-NMR of compound **1**.

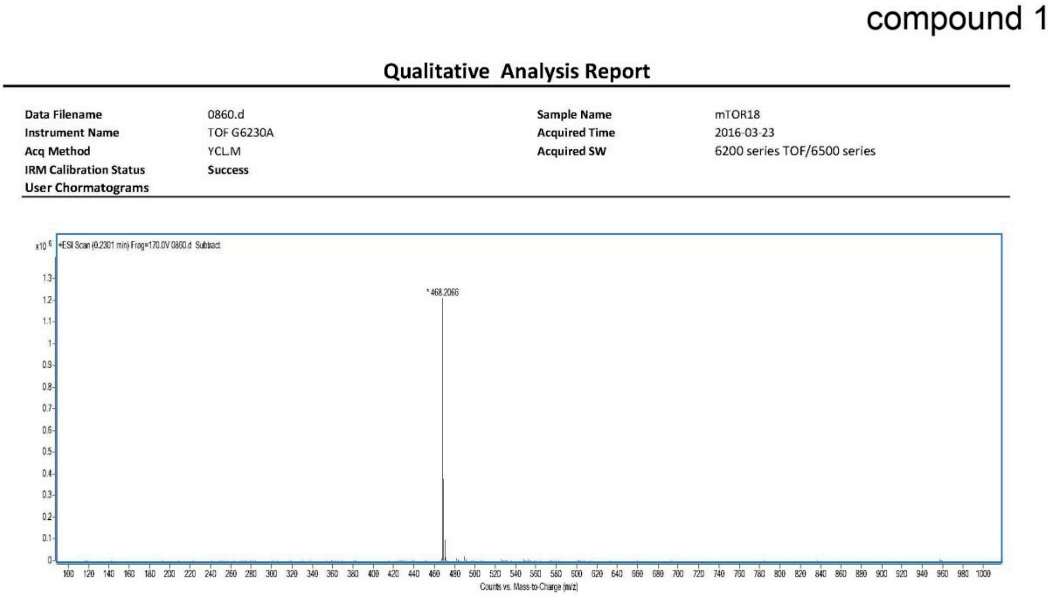

Figure S3. HRMS of compound **1**.

Ethyl 4-(6-(4-(3-ethylureido)phenyl)-4-morpholinobenzo[d]thiazol-2-yl) piperazine-1-carboxylate (**2**)

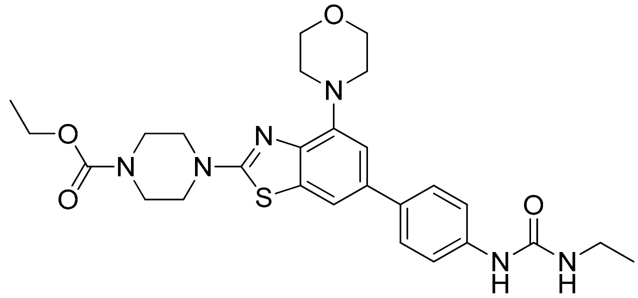

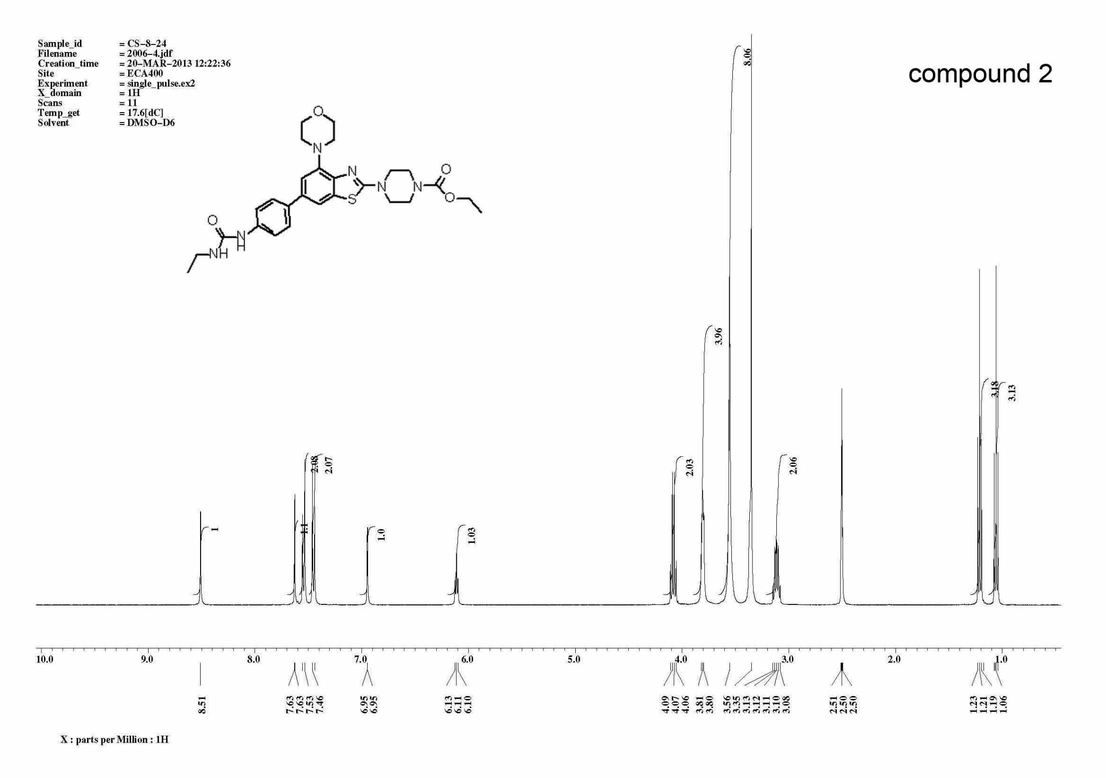

Figure S4. ^{1}H-NMR of compound **2**.

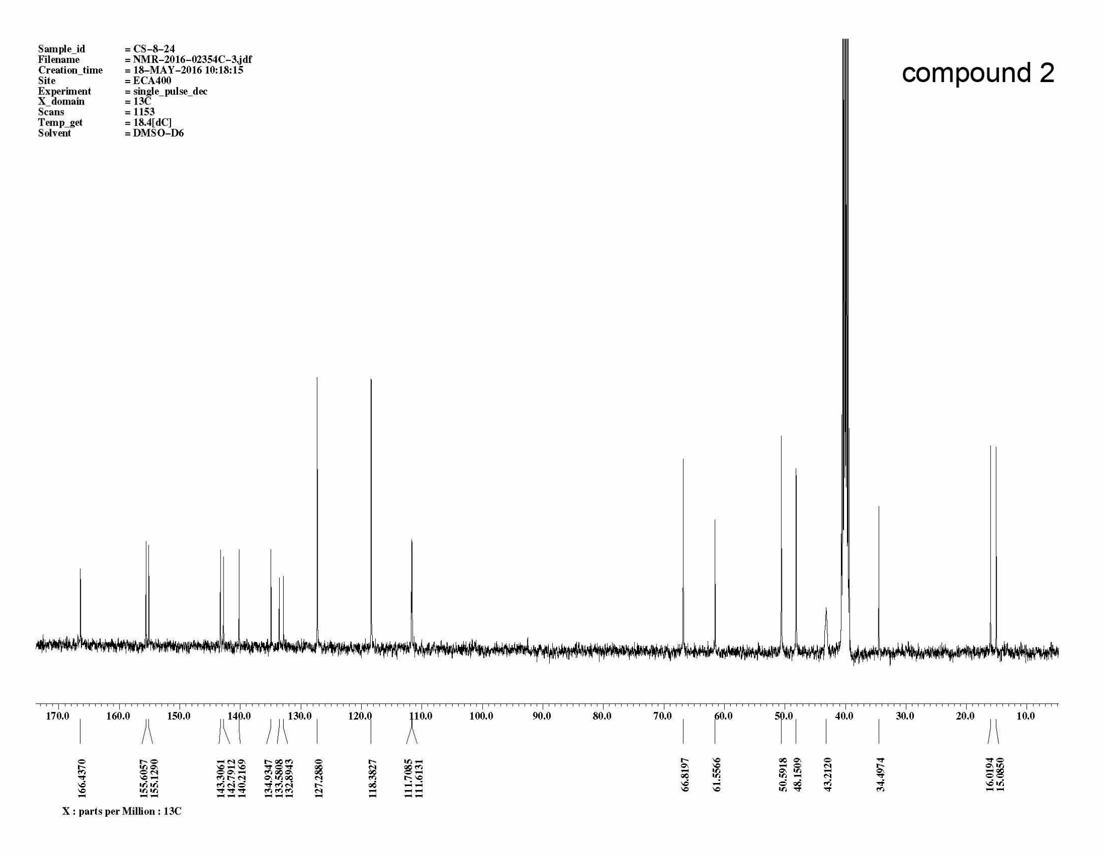

Figure S5. ^{13}C-NMR of compound **2**.

compound 2

Qualitative Analysis Report

**Data Filename**	1416.d	**Sample Name**	CS-8-24
**Instrument Name**	TOF G6230A	**Acquired Time**	2016-05-24
**Acq Method**	YCL.M	**Acquired SW**	6200 series TOF/6500 series
**IRM Calibration Status**	Success		

User Chormatograms

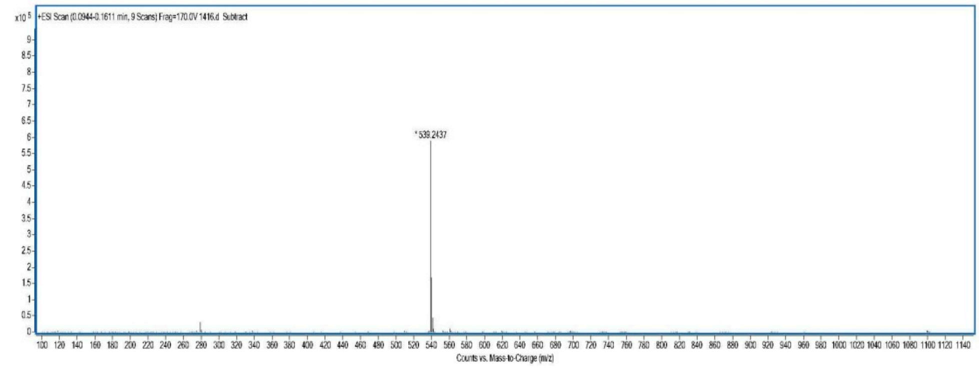

Figure S6. HRMS of compound **2**.

1-Ethyl-3-(4-(2-(4-isopropylpiperazin-1-yl)-4-morpholinobenzo[d]thiazol-6-yl)phenyl)urea (**3**)

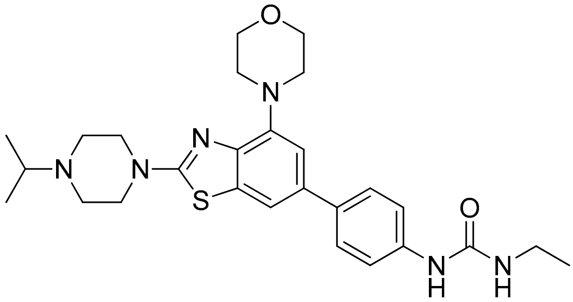

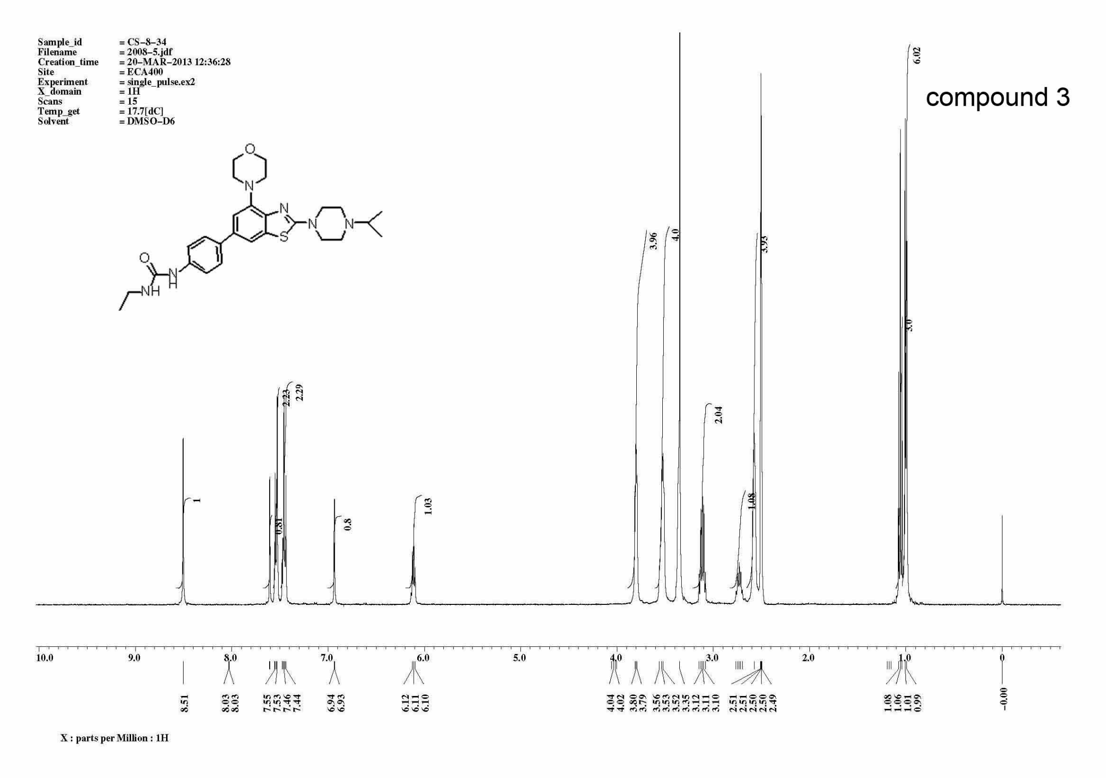

Figure S7. ^{1}H-NMR of compound **3**.

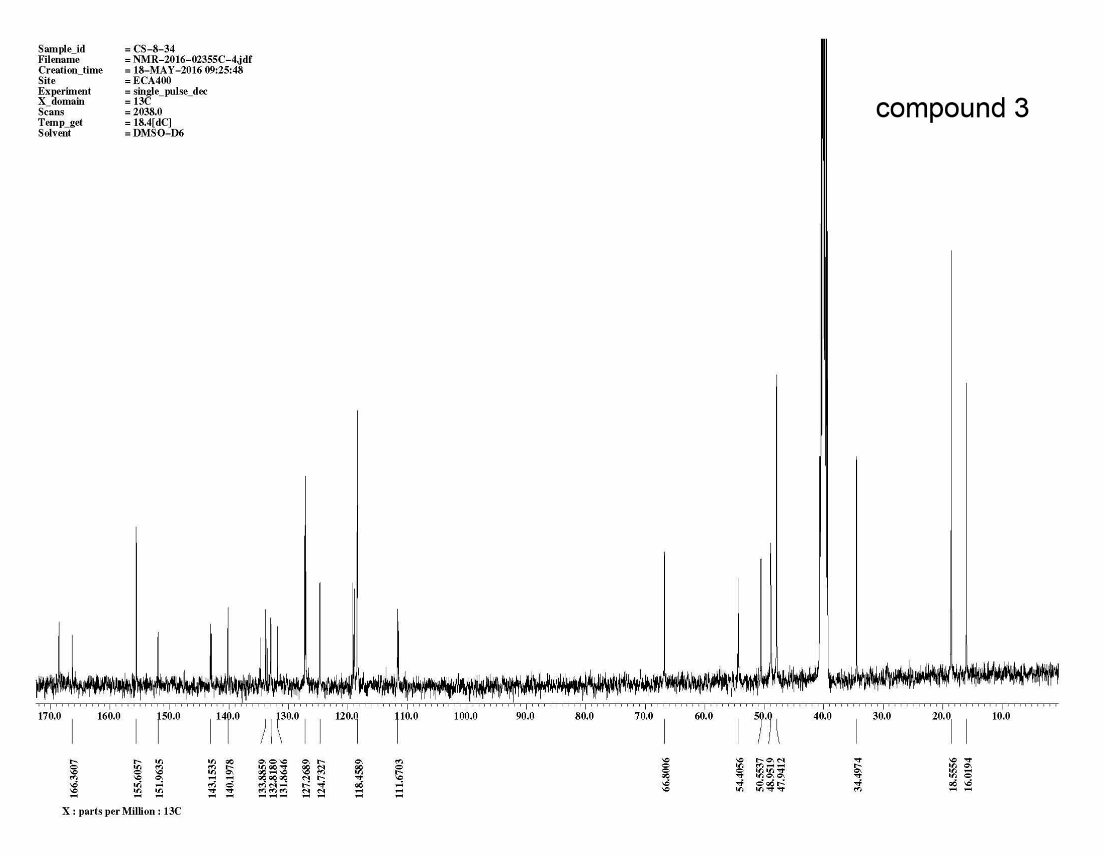

Figure S8. ^{13}C-NMR of compound **3**.

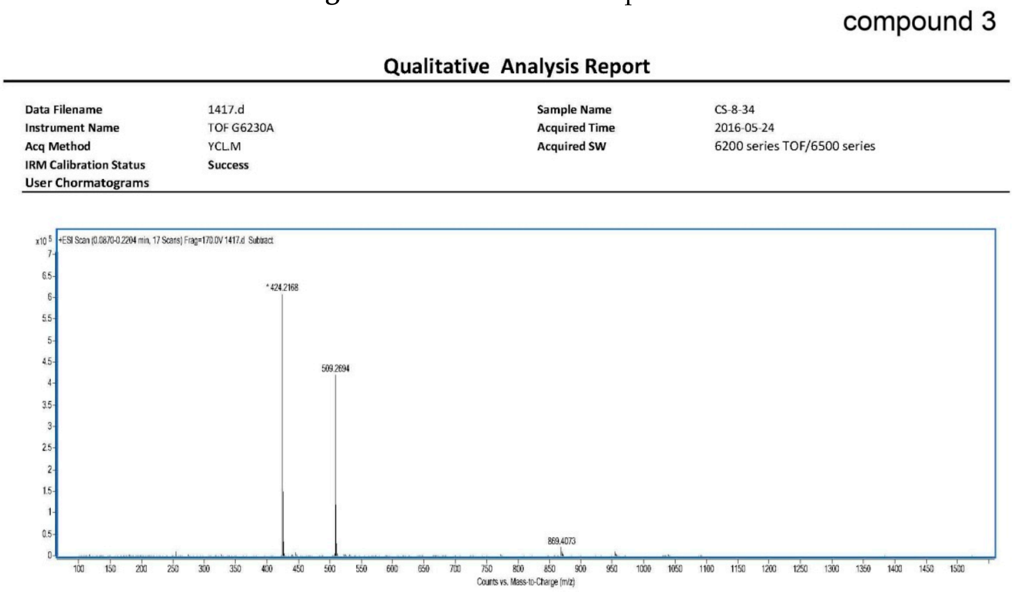

Figure S9. HRMS of compound **3**.

1-Ethyl-3-(4-(4-morpholino-2-(piperidin-1-yl)benzo[d]thiazol-6-yl)phenyl)urea (**4**)

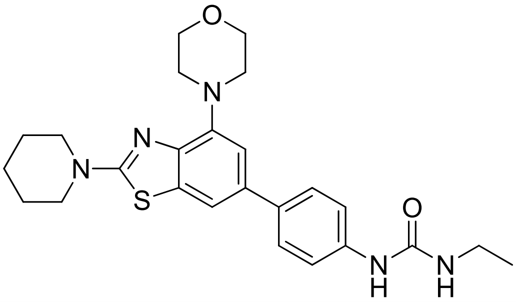

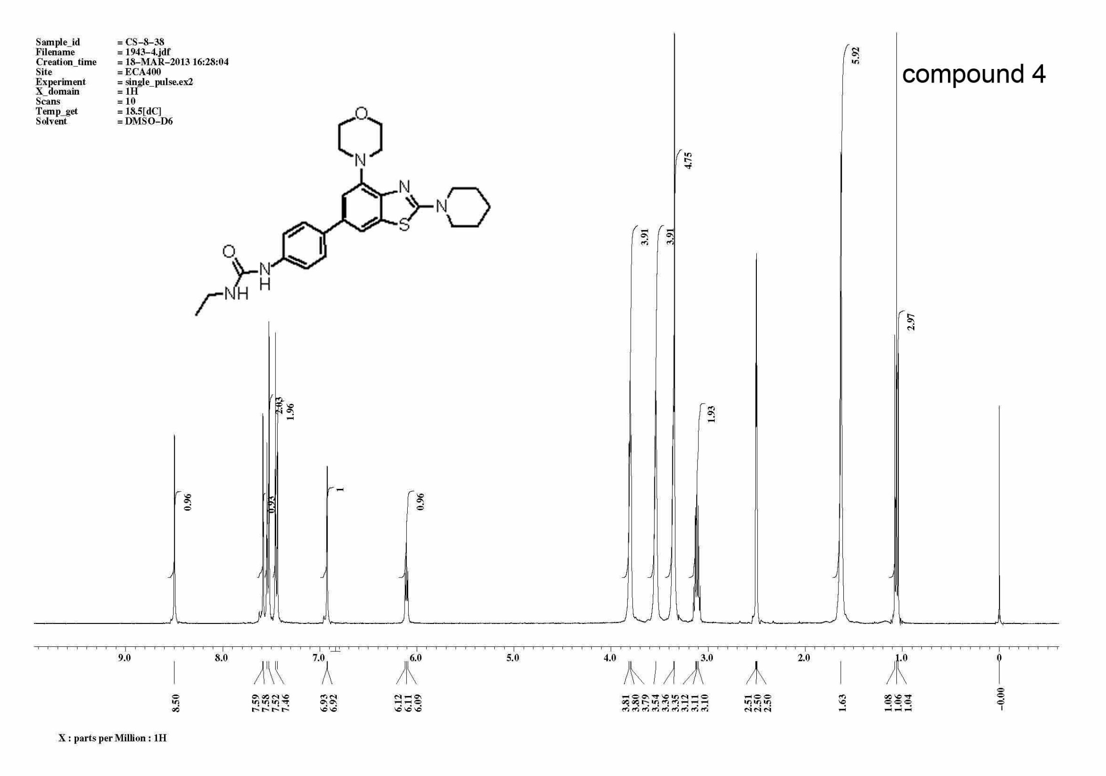

Figure S10. ^{1}H-NMR of compound **4**.

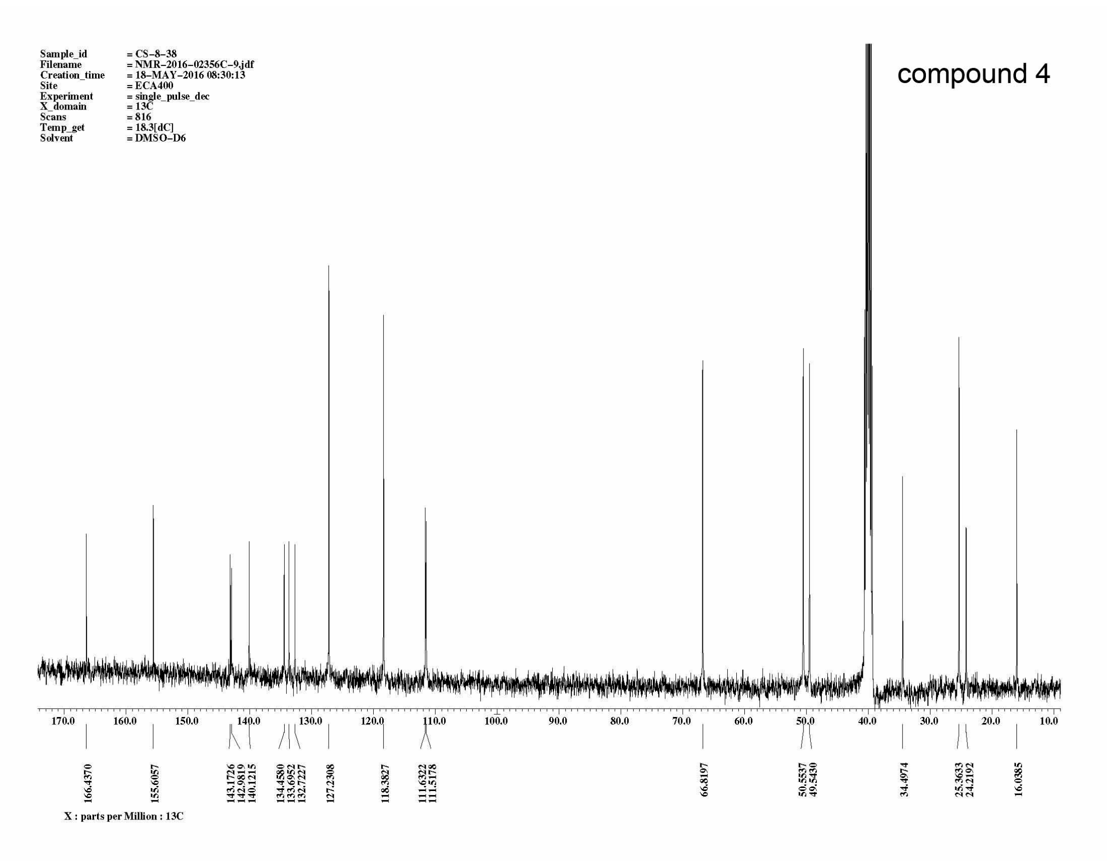

Figure S11. ^{13}C-NMR of compound **4**.

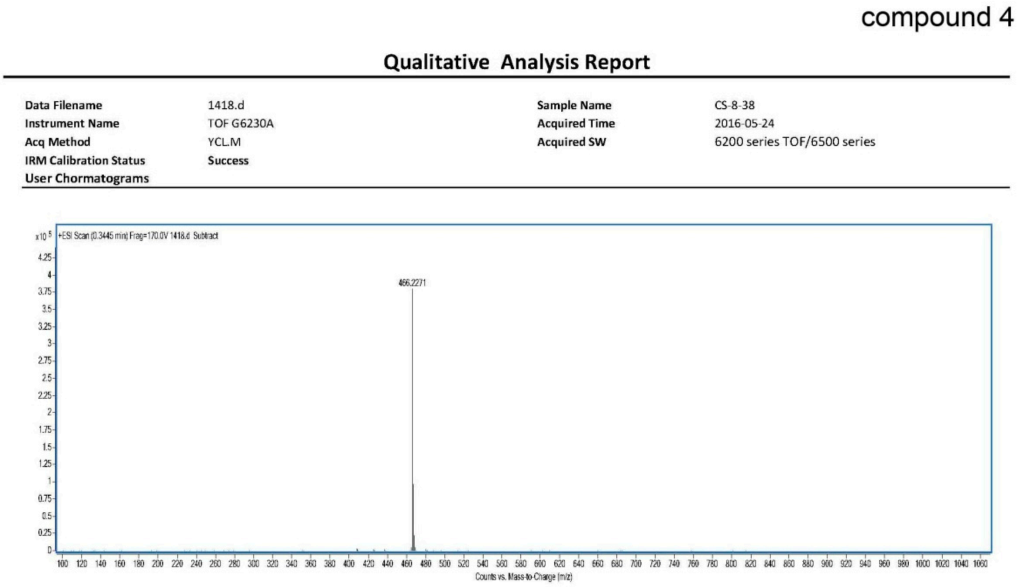

Figure S12. HRMS of compound **4**.

1-Cyclopropyl-3-(4-(2,4-dimorpholinobenzo[d]thiazol-6-yl)phenyl)urea (**5**)

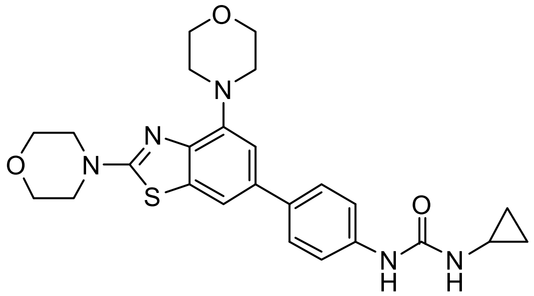

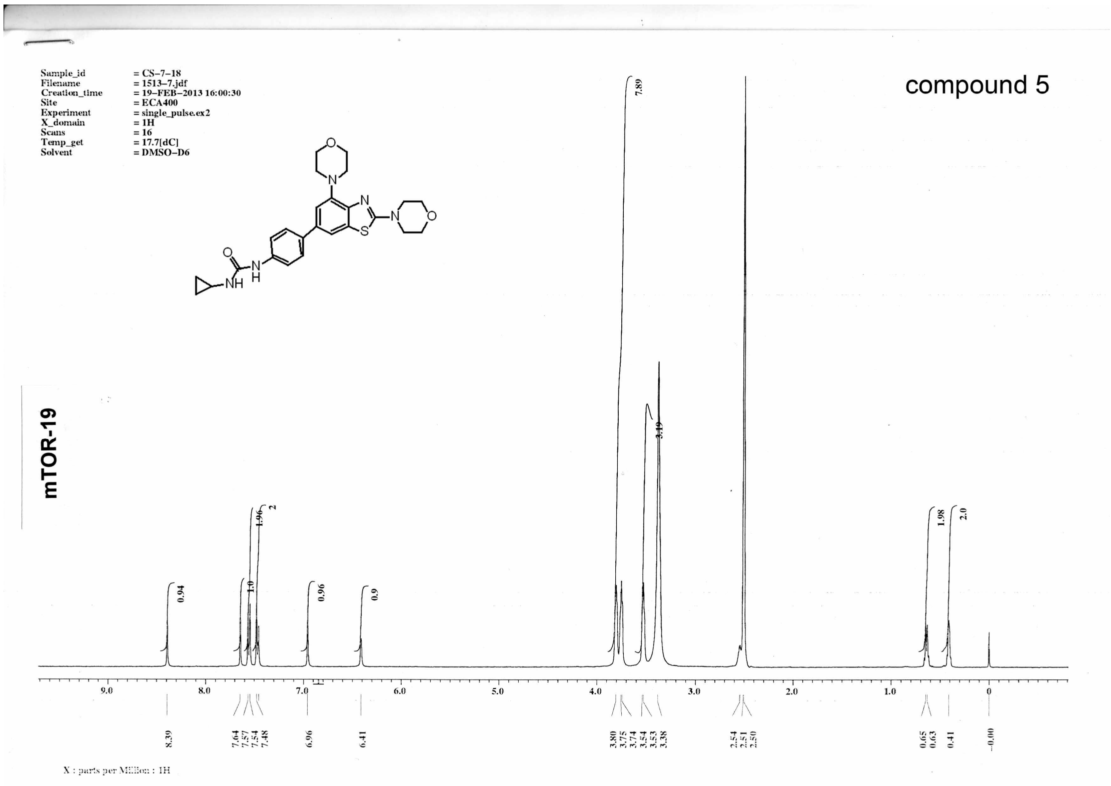

Figure S13. ^{1}H-NMR of compound **5**.

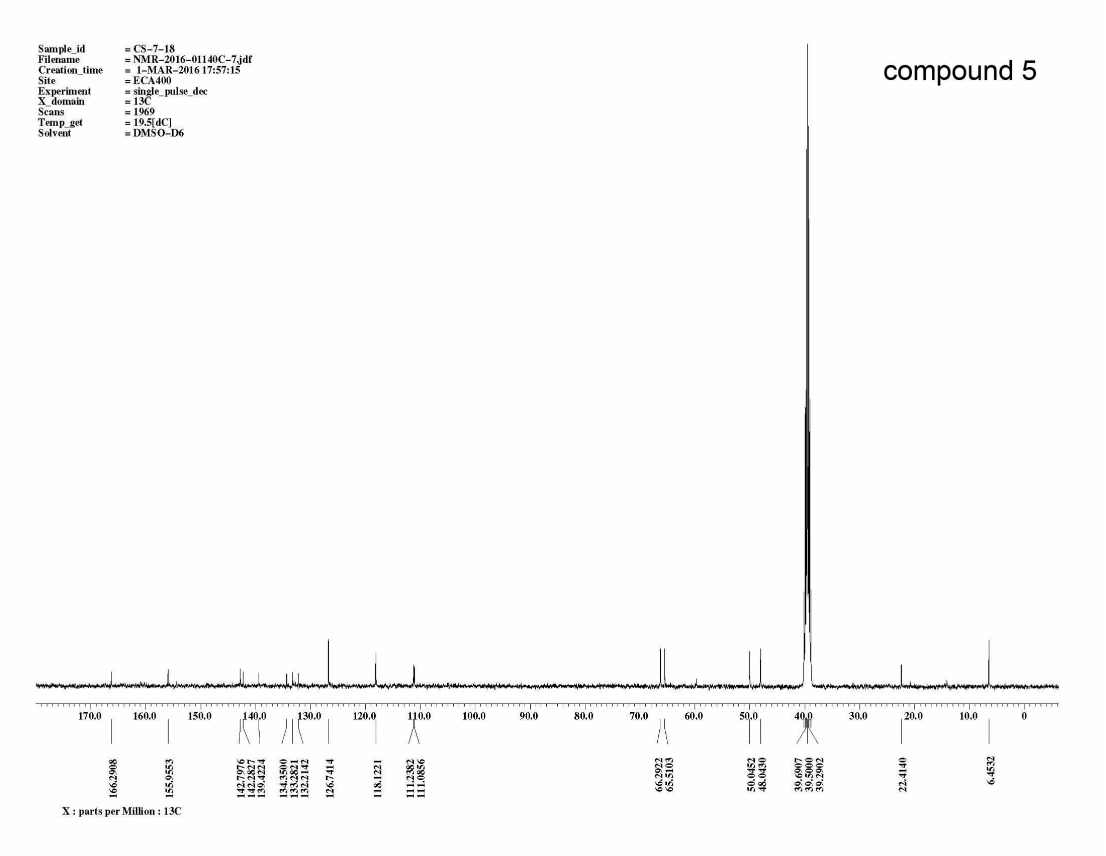

Figure S14. ^{13}C-NMR of compound **5**.

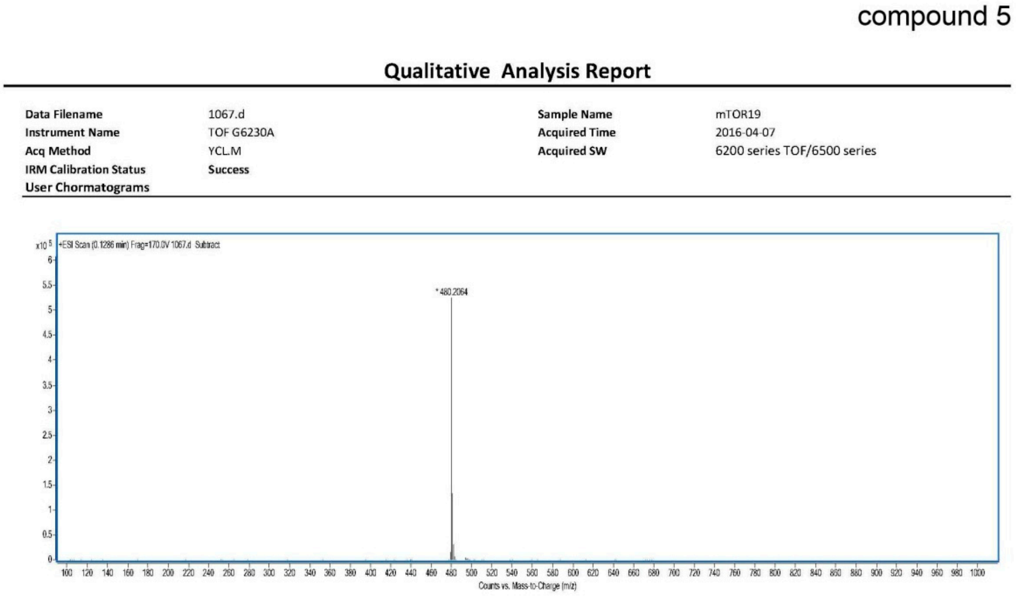

Figure S15. HRMS of compound **5**.

1-Benzyl-3-(4-(2,4-dimorpholinobenzo[d]thiazol-6-yl)phenyl)urea (**6**)

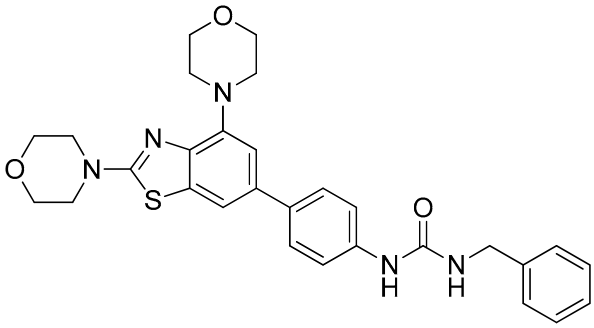

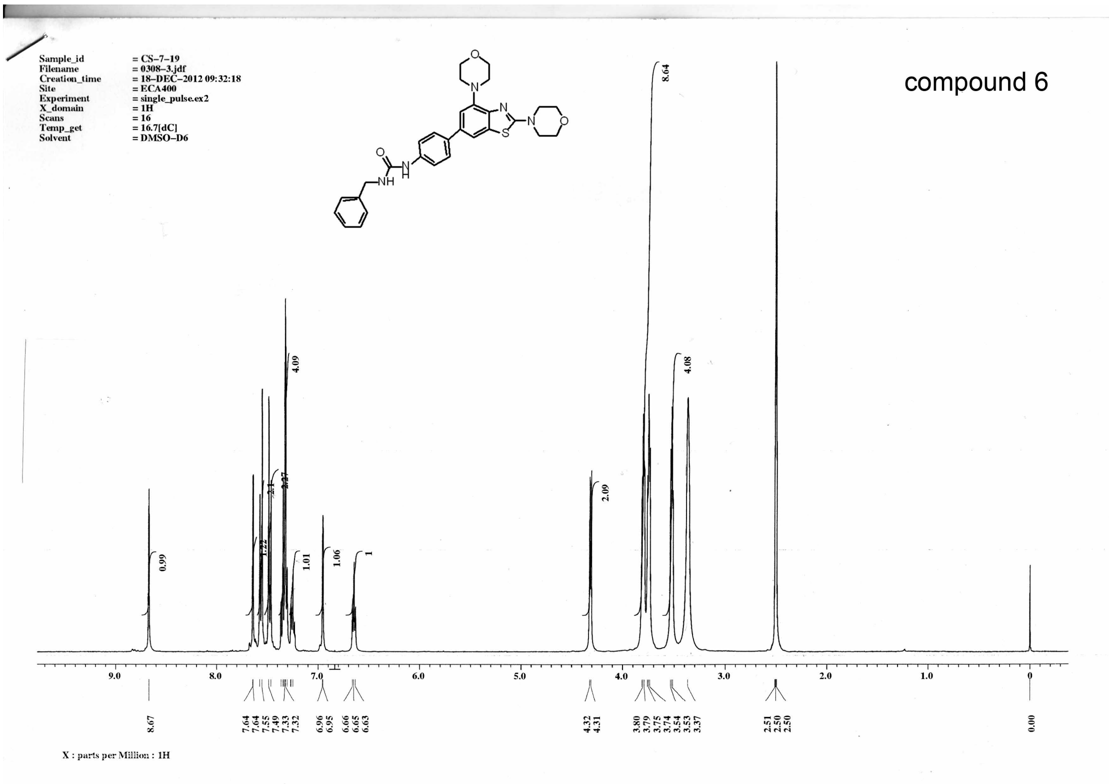

Figure S16. ^{1}H-NMR of compound **6**.

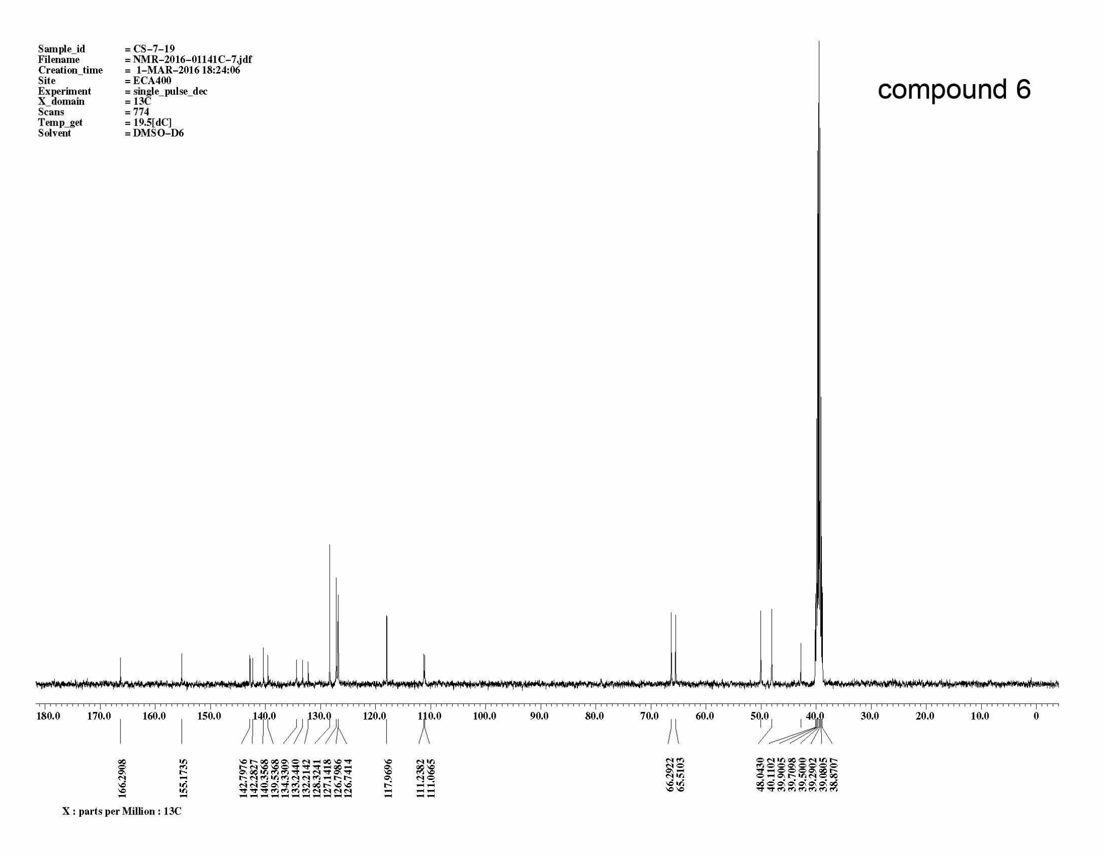

Figure S17. ^{13}C-NMR of compound **6**.

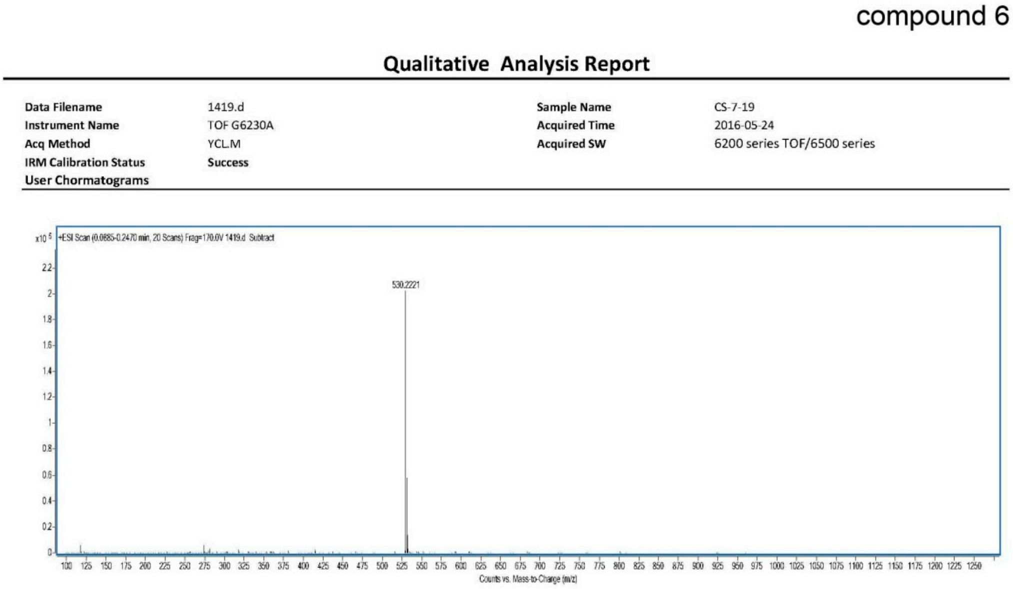

Figure S18. HRMS of compound **6**.

1-(2,4-Difluorophenyl)-3-(4-(2,4-dimorpholinobenzo[d]thiazol-6-yl)phenyl)urea (**7**)

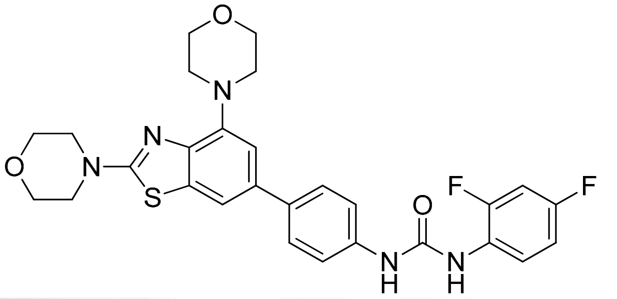

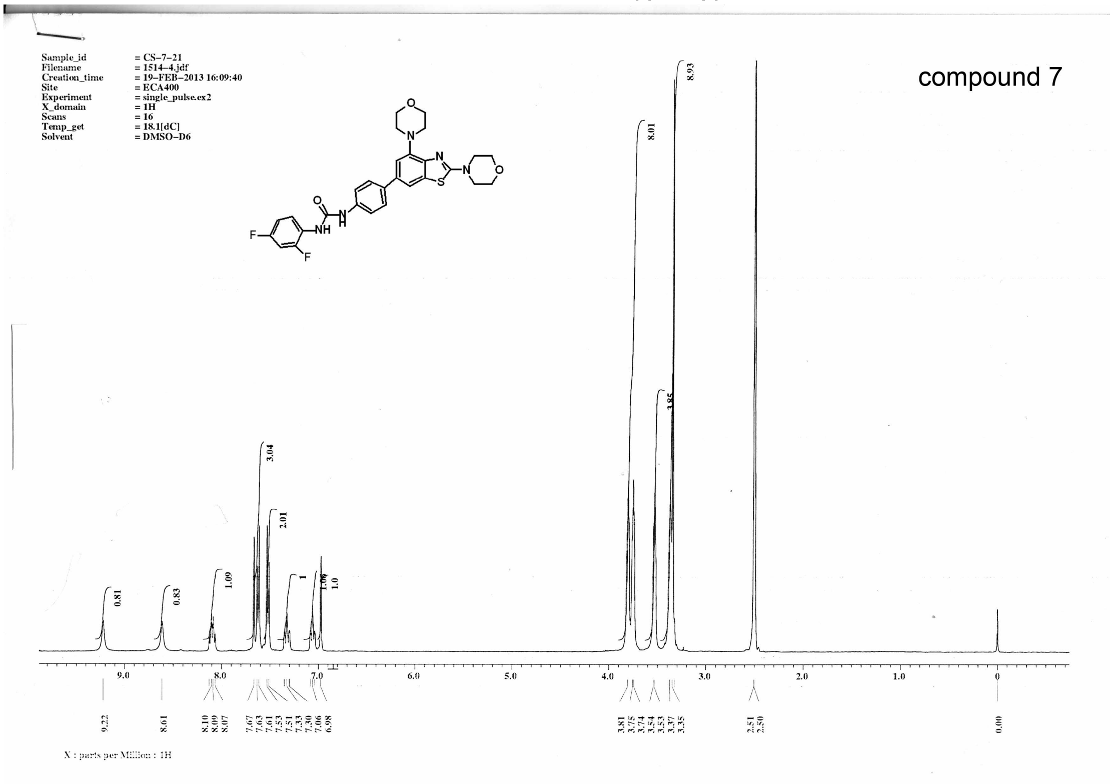

Figure S19. ^{1}H-NMR of compound **7**.

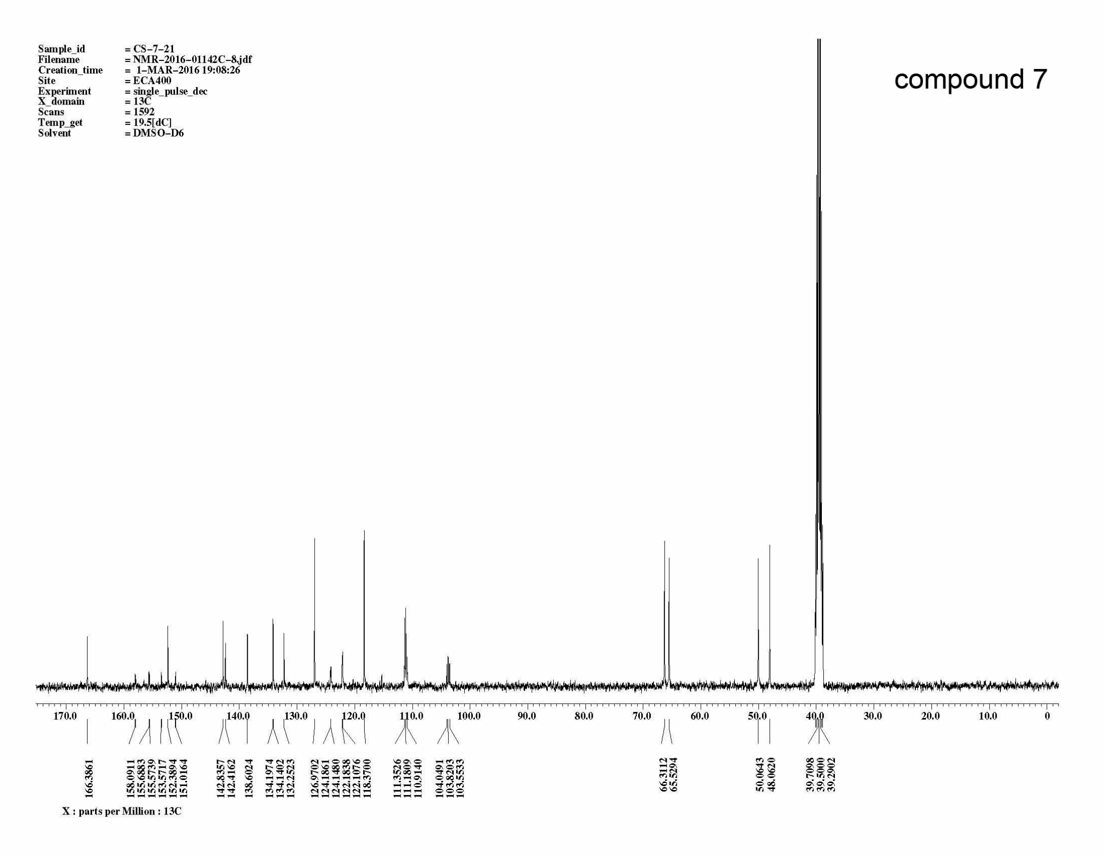

Figure S20. ^{13}C-NMR of compound **7**.

compound 7

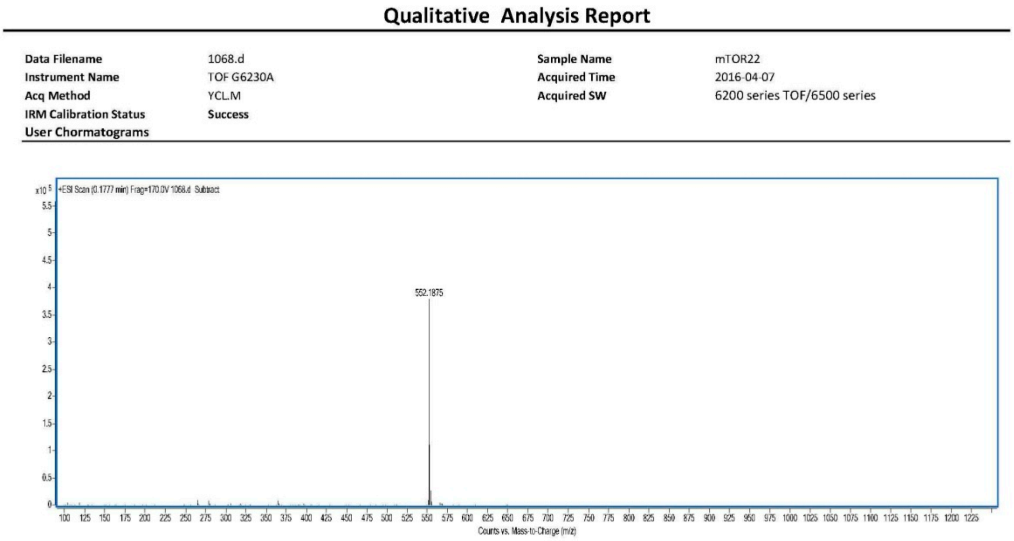

Figure S21. HRMS of compound **7**.

1-(4-(2,4-Dimorpholinobenzo[d]thiazol-6-yl)phenyl)-3-methylurea (**8**)

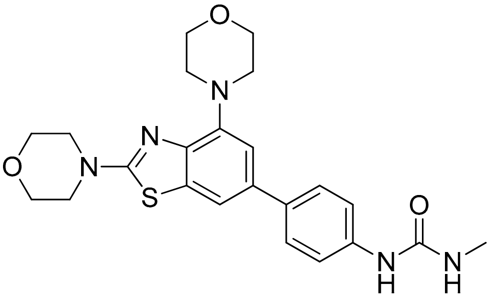

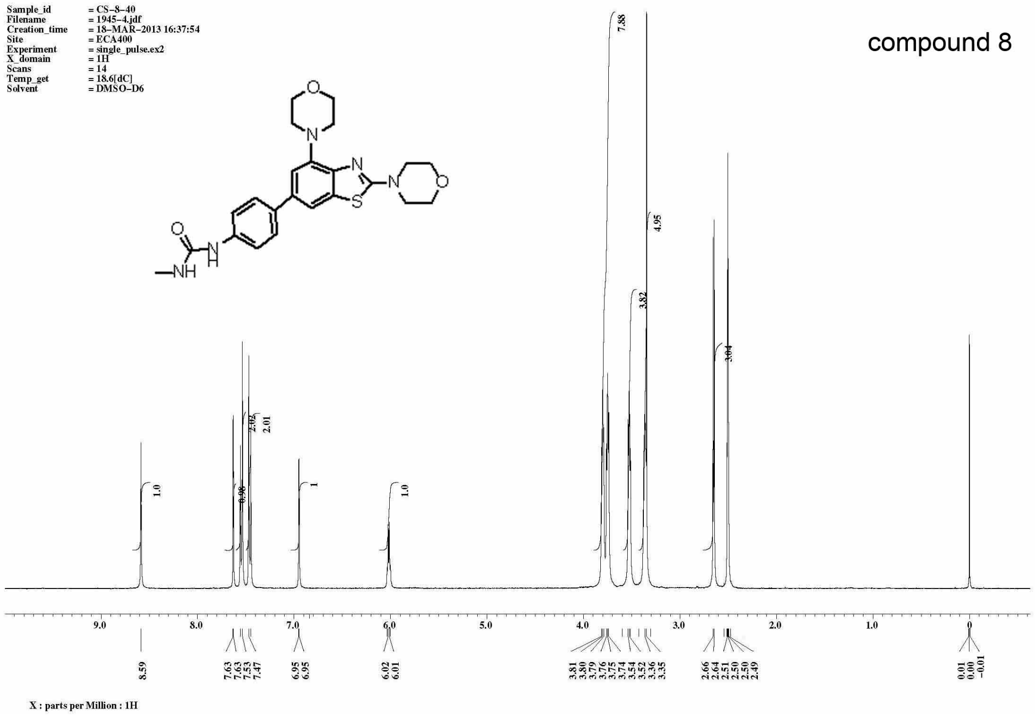

Figure S22. ^{1}H-NMR of compound **8**.

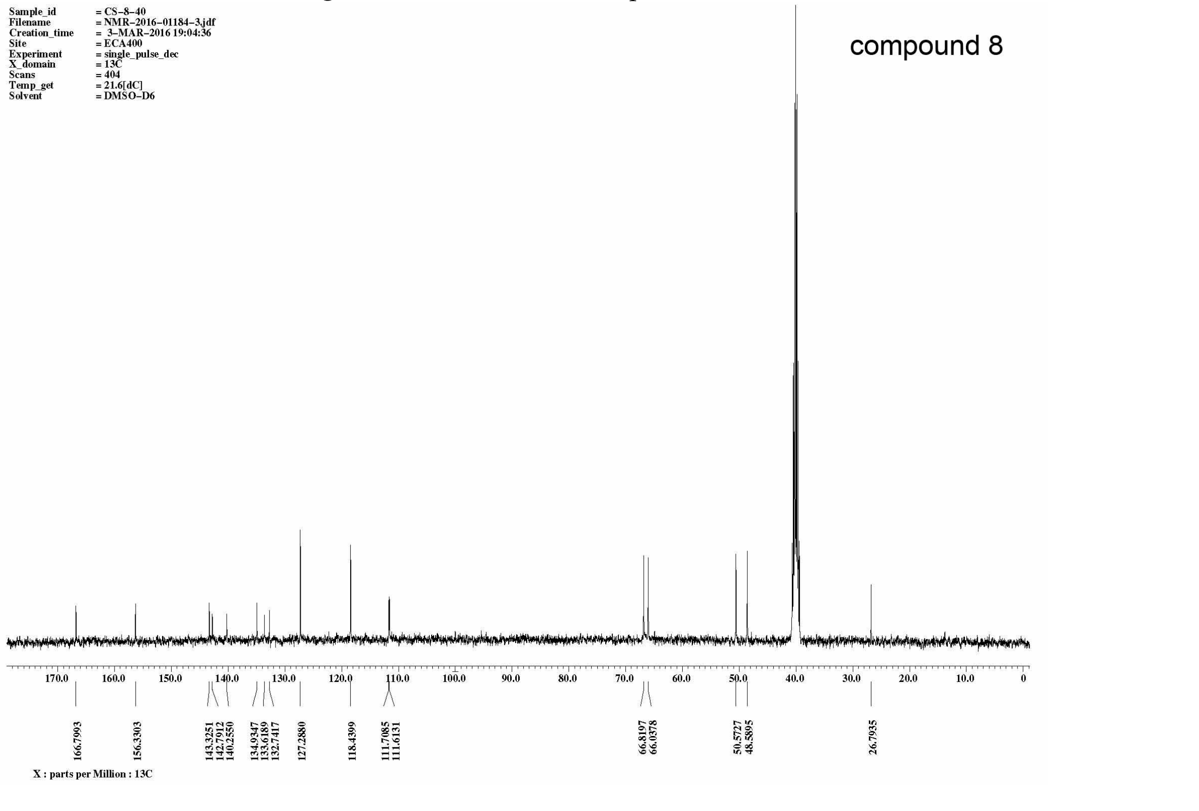

Figure S23. ^{13}C-NMR of compound **8**.

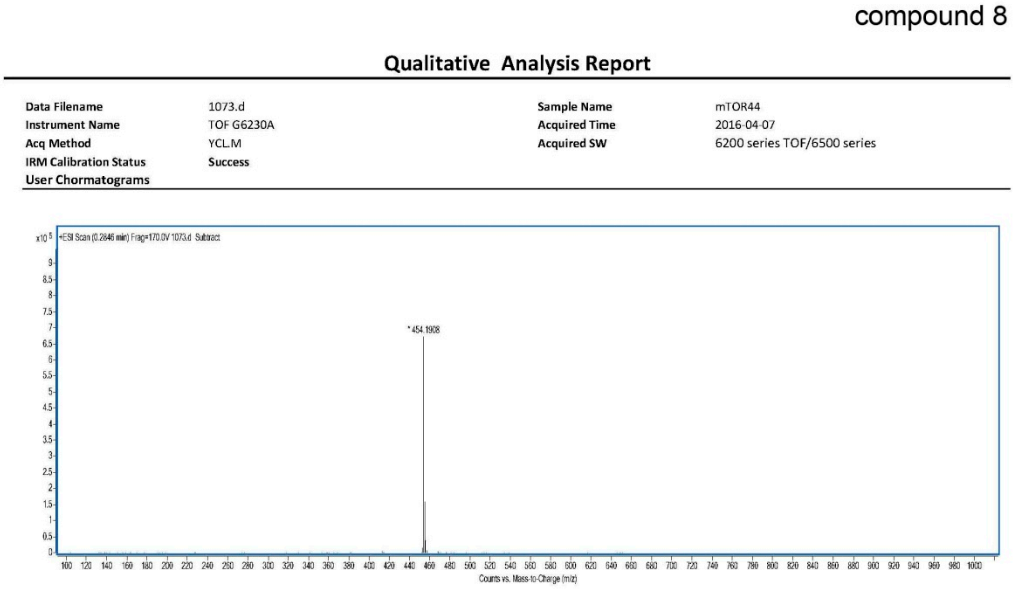
compound 8

Qualitative Analysis Report

**Data Filename**	1073.d	**Sample Name**	mTOR44
**Instrument Name**	TOF G6230A	**Acquired Time**	2016-04-07
**Acq Method**	YCL.M	**Acquired SW**	6200 series TOF/6500 series
**IRM Calibration Status**	Success		
**User Chormatograms**			

Figure S24. HRMS of compound **8**.

1-(4-(2,4-Dimorpholinobenzo[d]thiazol-6-yl)phenyl)-3-(4-(4-methylpiperazin-1-yl)phenyl)urea (**9**)

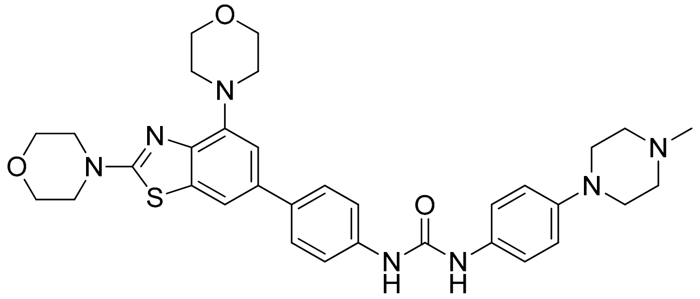

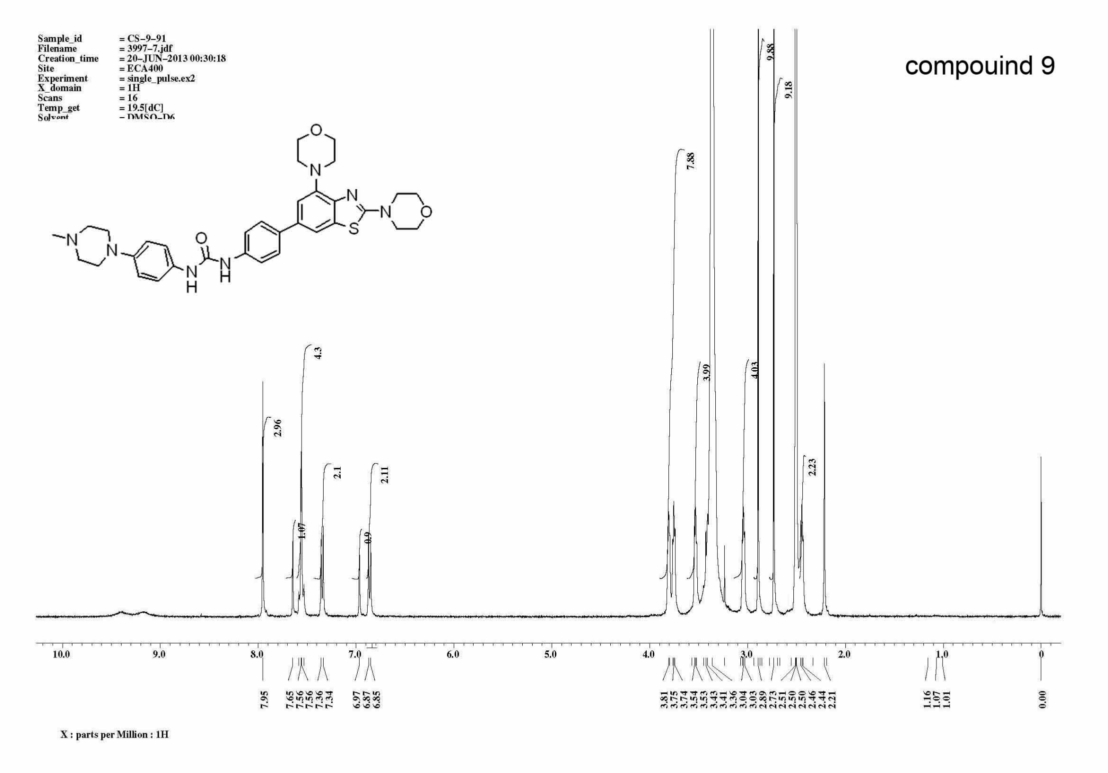

Figure S25. ^{1}H-NMR of compound **9**.

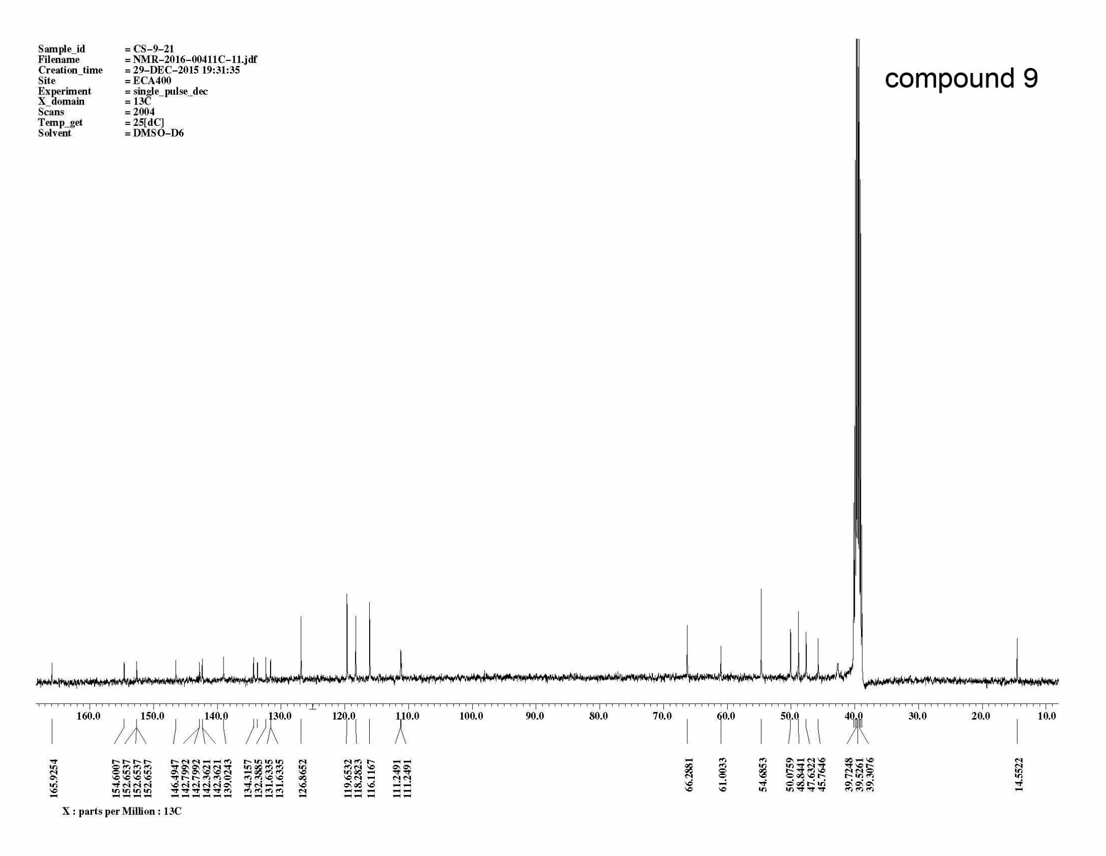

Figure S26. ^{13}C-NMR of compound **9**.

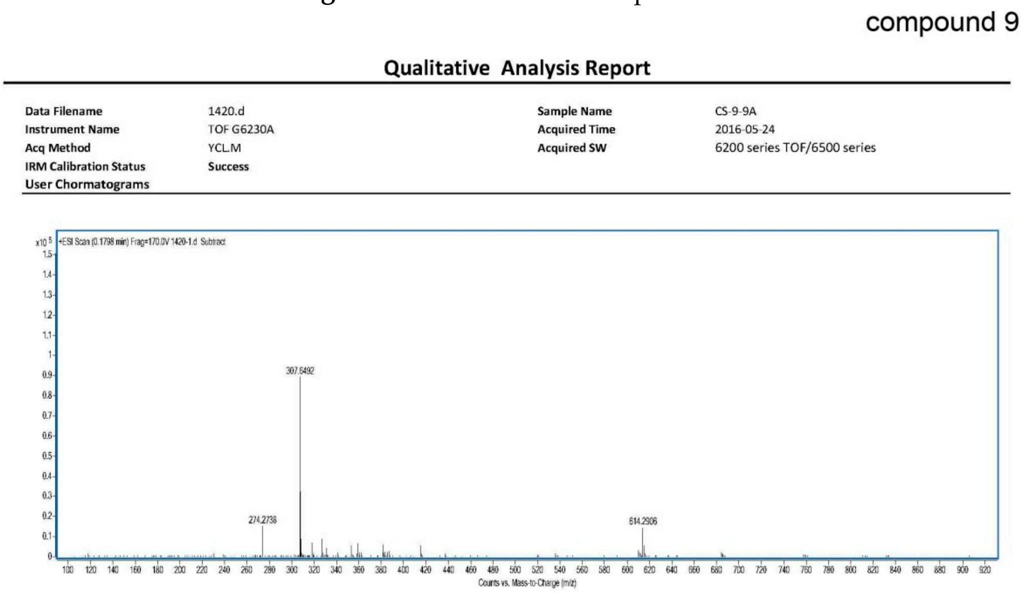

Figure S27. HRMS of compound **9**.

1-(4-(2,4-Dimorpholinobenzo[d]thiazol-6-yl)phenyl)-3-(4-(hydroxymethyl)phenyl)urea (**10**)

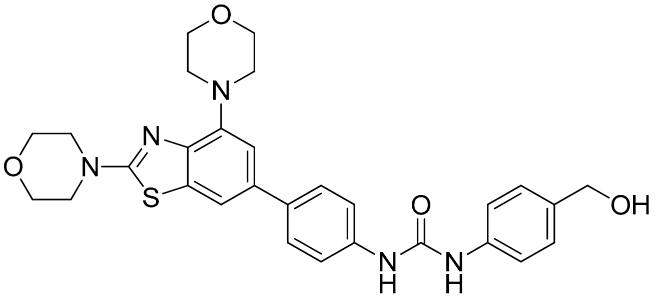

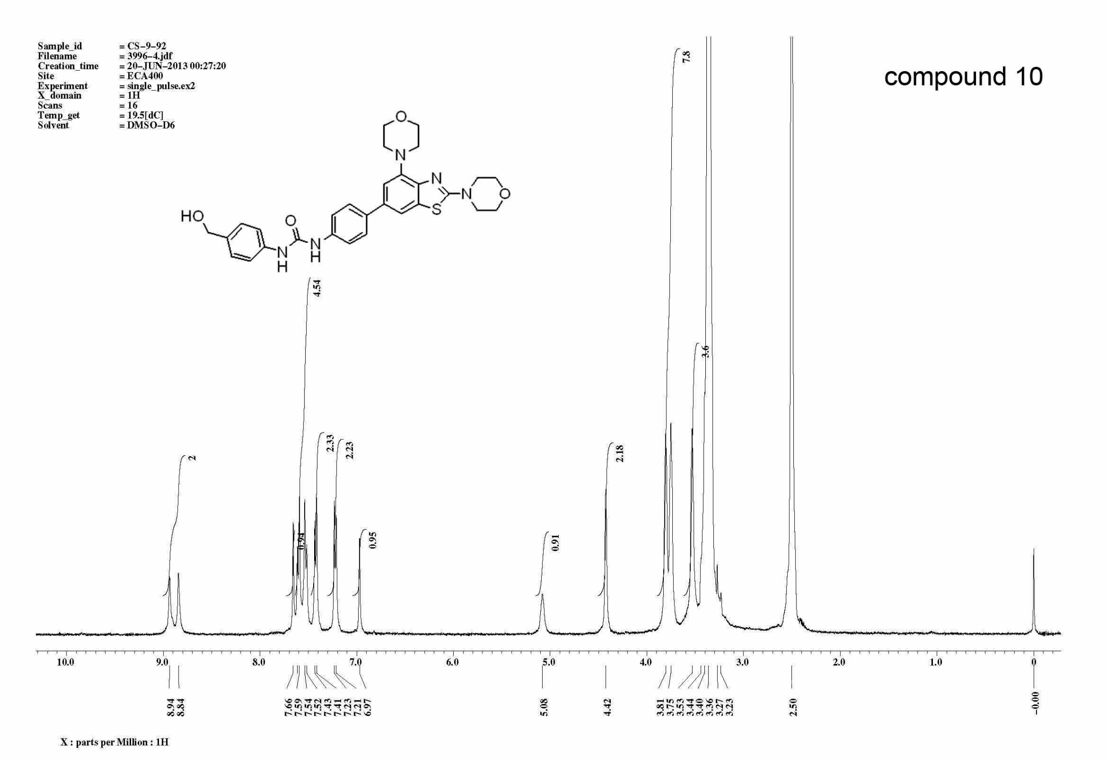

Figure S28. ^{1}H-NMR of compound **10**.

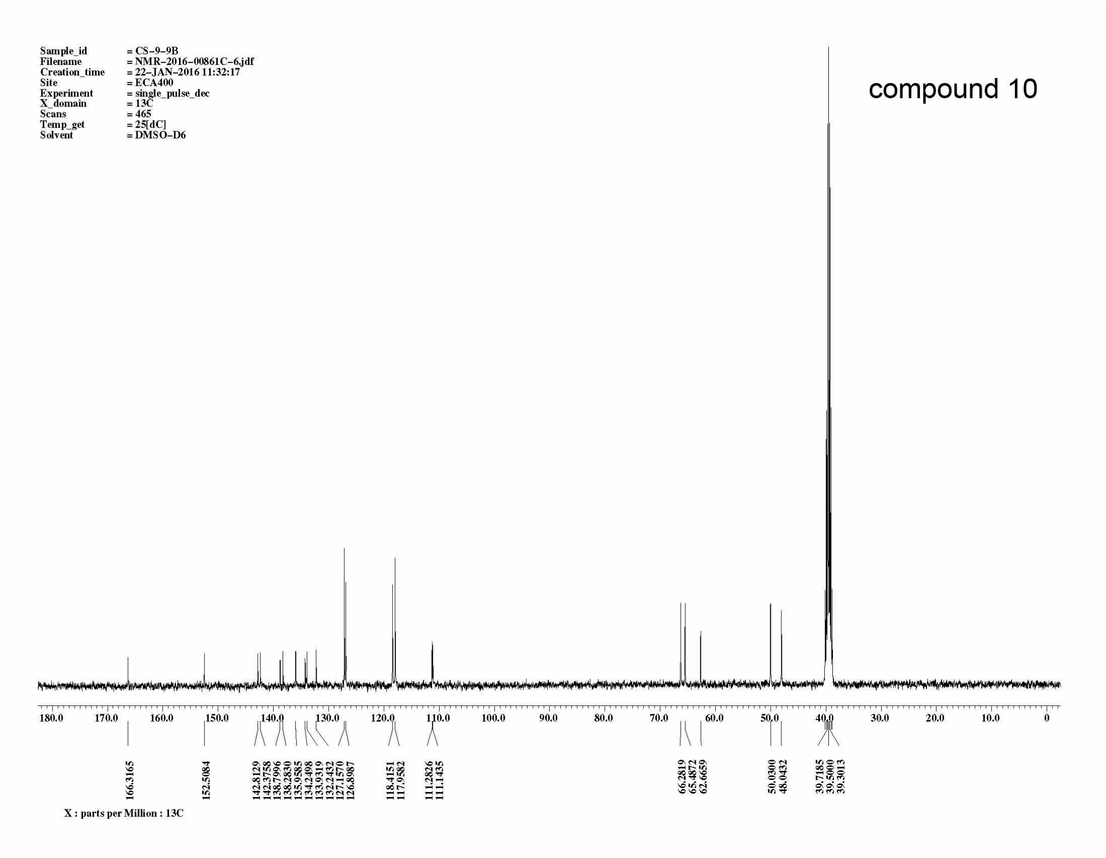

Figure S29. ^{13}C-NMR of compound **10**.

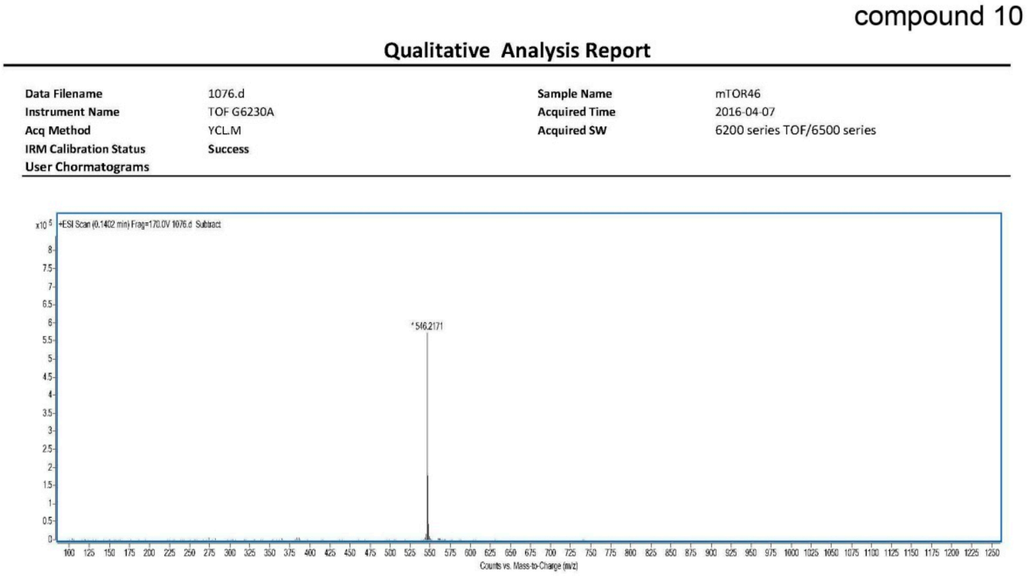

Figure S30. HRMS of compound **10**.

1-(4-(2,4-Dimorpholinobenzo[d]thiazol-6-yl)phenyl)-3-(pyridin-3-yl)urea (**11**)

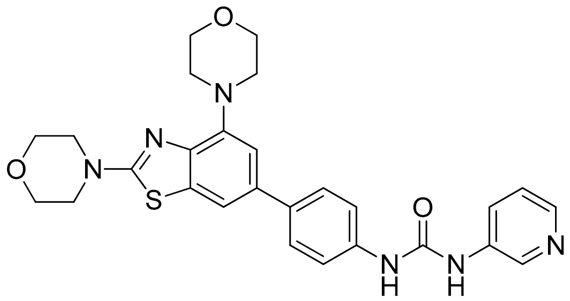

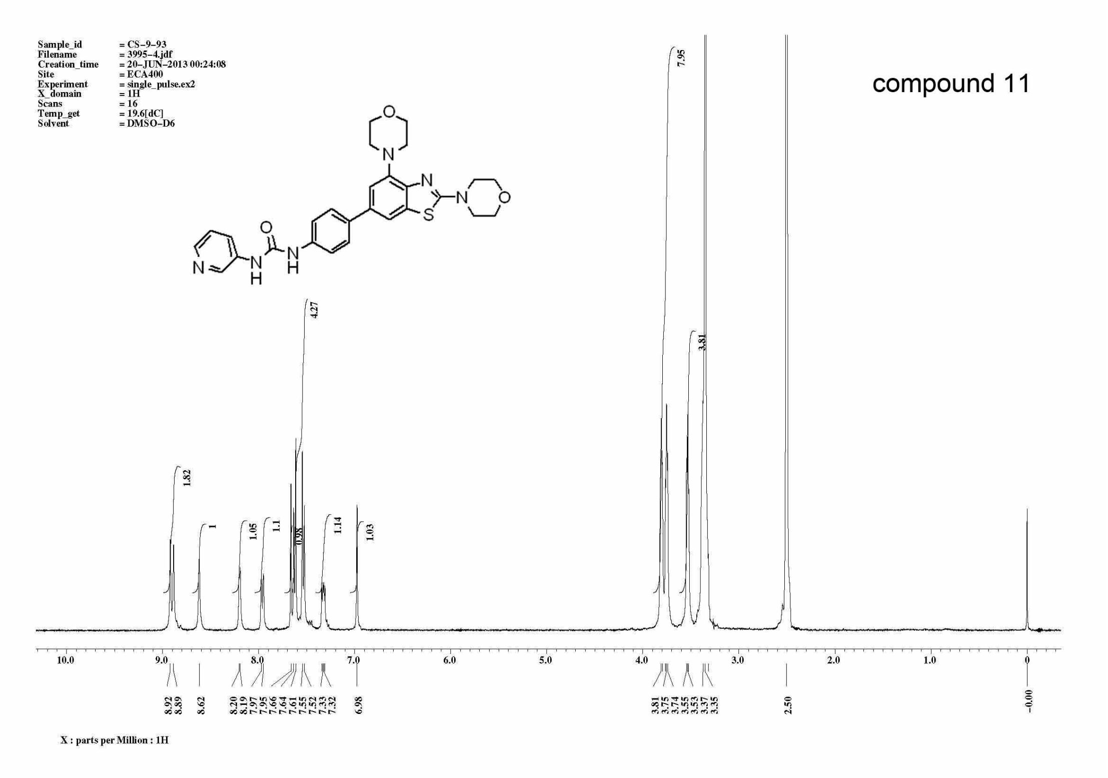

Figure S31. ^{1}H-NMR of compound **11**.

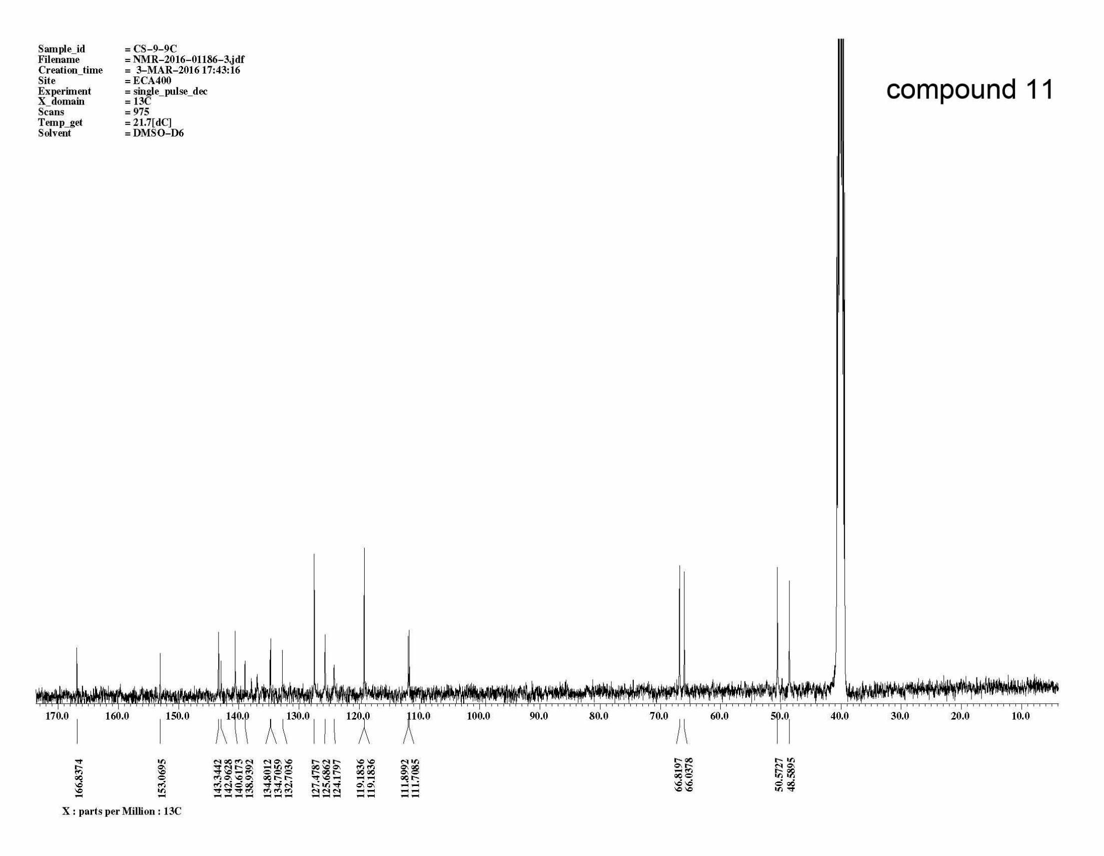

Figure S32. ^{13}C-NMR of compound **11**.

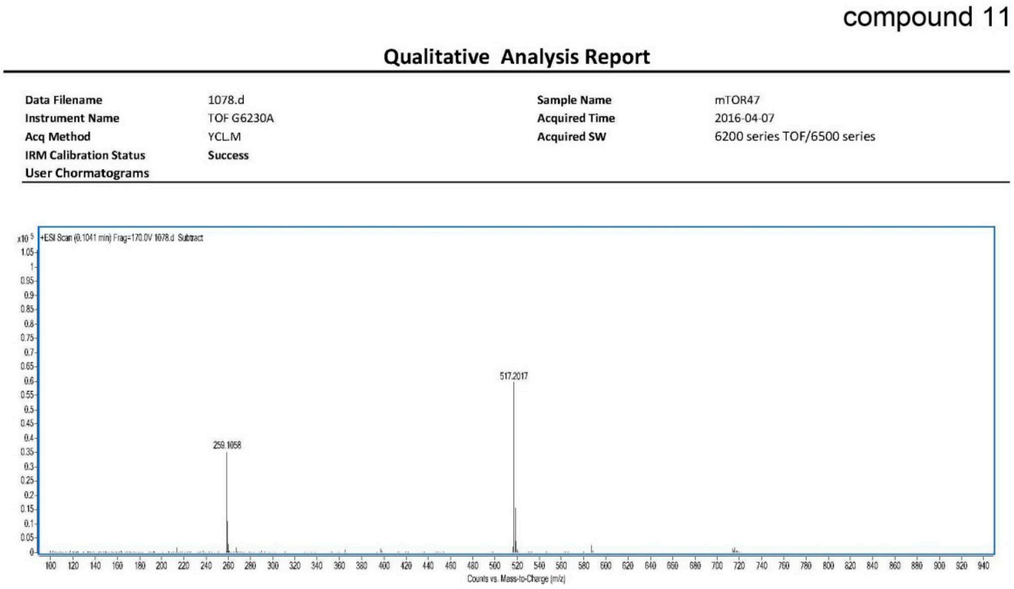

Figure S33. HRMS of compound **11**.
